# Supplementary material for: Understanding the inclusion and participation of adults from Black African Diaspora Communities (BAFDC) in health and care research in the UK: a realist review protocol
Source: BMJ Open. 2024 Mar 29;14(3):e082564. doi: 10.1136/bmjopen-2023-082564 (PMC10982753; doi:10.1136/bmjopen-2023-082564)
Supplement: Supplementary data [file bmjopen-2023-082564supp001.pdf]

Supplementary file 1. Positionality statement

The authorship team is based in the UK and consists of individuals from the following backgrounds: academic, clinical, clinical academic, leaders of community organisations, health and social care research delivery, health and social care research funding and leaders of third sector organisations, (government and voluntary). All authors have consented to self-reporting their ethnicity, as indicated on the title page. Some have experience of conducting research, delivering research, funding research, providing healthcare, or support and representation in some way, to people from Black African Diaspora Communities (BAFDC). The co-production group (VE, TK, DG, LH, EM,TO, IS, SW) and one member of the research team (DE) have lived experience of being Black, as defined in the protocol. Two members of the co-production group identify as Black men.The first author (EH) is a White British clinical academic researcher whose work mainly focuses on developing an inclusive health and care research system for individuals from BAFDC. Over the last ten years, she has observed the exclusionary practices, processes and service provision of health and social care research to people from BAFDC through her role as a clinical research nurse in the West Midlands region of England. She has been actively involved as a Patient Public Involvement Lead, contributing to her awareness of the impact of exclusion from health and social care research on the health and well-being of people from BAFDC as well as the difficulties they face in opportunities to take part in health and social care research. She and all co-authors acknowledge the importance of equity, diversity and inclusion, and value an inclusive approach to health and social care research where all individuals, including those from under-served communities, benefit from research opportunities. They will be reflexive throughout this research on their positionality and how this may influence interpretations of people’s lived experiences. All authors work as a team and the first author (EH) has regular discussions with the co-production group to ensure the protocol and subsequent review will be led by their cultural and experiential knowledge and expertise.
