# Supplementary material for: Understanding the inclusion and participation of adults from Black African Diaspora Communities (BAFDC) in health and care research in the UK: a realist review protocol
Source: BMJ Open. 2024 Mar 29;14(3):e082564. doi: 10.1136/bmjopen-2023-082564 (PMC10982753; doi:10.1136/bmjopen-2023-082564)
Supplement: Supplementary data [file bmjopen-2023-082564supp002.pdf]

Ovid MEDLINE(R) Example Search Strategy

1	exp Clinical Trials as Topic/ or clinical trial*.mp.
2	exp Health Services Research/ or exp Biomedical Research/
3	exp African Americans/
4	black british.mp.
5	participation.mp. or Patient Participation/ or Community Participation/ or Stakeholder Participation/ or Social Participation/
6	inclusion.mp.
7	recruitment.mp.
8	(underrepresent* or under-represent*).mp.
9	Black people.mp. or Black People/
10	Afro-Caribbean.mp.
11	BME.mp.
12	BAME.mp.
13	1 or 2
14	3 or 4 or 9 or 10 or 11 or 12
15	5 and 13 and
16	6 or 7 or 8
17	5 or 16
18	13 and 14 and 16
19	1 and 5 and 14
20	1 and 14 and 17
