# Supplementary material for: Understanding the inclusion and participation of adults from Black African Diaspora Communities (BAFDC) in health and care research in the UK: a realist review protocol
Source: BMJ Open. 2024 Mar 29;14(3):e082564. doi: 10.1136/bmjopen-2023-082564 (PMC10982753; doi:10.1136/bmjopen-2023-082564)
Supplement: Author's manuscript [file bmjopen-2023-082564.draft_revisions.pdf]

BMJ Open

BMJ Open is committed to open peer review. As part of this commitment we make the peer review history of every article we publish publicly available.

When an article is published we post the peer reviewers' comments and the authors' responses online. We also post the versions of the paper that were used during peer review. These are the versions that the peer review comments apply to.

The versions of the paper that follow are the versions that were submitted during the peer review process. They are not the versions of record or the final published versions. They should not be cited or distributed as the published version of this manuscript.

BMJ Open is an open access journal and the full, final, typeset and author-corrected version of record of the manuscript is available on our site with no access controls, subscription charges or pay-per-view fees (http://bmjopen.bmj.com).

If you have any questions on BMJ Open's open peer review process please email info.bmjopen@bmj.com

BMJ Open

Understanding the inclusion and participation of adults from Black African Diaspora Communities (BAFDC) in health and social care research in the UK: a rapid realist review protocol.

Journal:	*BMJ Open*
Manuscript ID	bmjopen-2023-082564
Article Type:	Protocol
Date Submitted by the Author:	27-Nov-2023
Complete List of Authors:	Hoverd, Eleanor; University of Warwick Medical School,<br>Effiom, Violet; NIHR Clinical Research Network West Midlands<br>Gravesande, Dionne; Public Contributor<br>Hollowood, Lorna; University of Worcester<br>Kelly, Tony; PPI, PPI Lead on Project<br>Mukuka, Esther; NIHR<br>Owatemi, Taiwo; UK Parliament<br>Sargeant, Ify; Public Contributor<br>Ward, Shane; Public Contributor<br>Spencer, Rachel; University of Warwick, Warwick Medical School<br>Edge, Dawn; Univ Manchester; The University of Manchester<br>Dale, Jeremy; University of Warwick, Warwick Medical School<br>Staniszewska, Sophie; University of Warwick, Warwick Medical School
Keywords:	Clinical Trial, Health Equity, Health Services, Health Services Accessibility, Health policy < HEALTH SERVICES ADMINISTRATION & MANAGEMENT, Patient Participation

SCHOLARONE™
Manuscripts

Title: Understanding the inclusion and participation of adults from Black African Diaspora Communities (BAFDC) in health and social care research in the UK: a rapid realist review protocol.

Corresponding Author: Eleanor Hoverd, White British, Unit of Academic Primary Care, Warwick Medical School, Warwick University, Gibbet Hill Rd, Coventry CV4 7AL, England, UK eleanor.hoverd@warwick.ac.uk

Co-authors:

Violet Effiom, Black, Clinical Research Network, West Midlands, UK

Dionne Gravesande, Black British of Caribbean Public Contributor, UK

Lorna Hollowood, Mixed heritage – Black Caribbean and White, School of Nursing and Midwifery, University of Worcester, UK

Tony Kelly, Black British-Caribbean Public Contributor, Birmingham, UK

Dr. Esther Mukuka, Black African (British) Head of Research Inclusion, National Institute for Health and Care Research (NIHR), UK

Taiwo Owatemi, British African, Labour MP, Coventry Northwest, UK

Dr. Ifeanyi Sargeant, Black African Public Contributor, Wrinehill, Staffordshire, UK

Shane Ward, Black Alkebulan/ African Public Contributor, Sandwell, UK

Associate Professor Rachel Spencer, White British, Unit of Academic Primary Care, University of Warwick, UK R.Spencer2@warwick.ac.uk

Professor Dawn Edge, African-Caribbean, Division of Psychology and Mental Health, University of Manchester, UK dawn.edge@manchester.ac.uk

Professor Jeremy Dale, White British, Unit of Academic Primary Care, Warwick Medical School, Warwick University, Coventry, UK jeremy.dale@warwick.ac.uk

Professor Sophie Staniszewska, White British of Polish origin, DPhil (Oxon), BSc (Hons), Warwick Research in Nursing, Warwick Medical School, Warwick University, Coventry, UK Sophie.Staniszewska@warwick.ac.uk

For peer review only

Abstract

INTRODUCTION

People from Black African Diaspora Communities (BAFDC) experience poorer health outcomes, have many long-term conditions and are persistently under-represented in health and social care research. There is limited focus on programmes, or interventions that support inclusion and participation of people from BAFDC in research. Through co-production, this realist review seeks to provide a programme theory that explains what context and mechanisms may be required, to produce outcomes that facilitate inclusion and participation for people from BAFDC in health and social care research in the UK.

METHODS AND ANALYSIS

A group of people from BAFDC with lived and professional experience representing all levels of the health and social care research system will co-produce a rapid realist review with a team of African-Caribbean, White British and White British of Polish origin health and care researchers. They will follow Pawson's five steps: 1) shaping the scope of the review; 2) searching for evidence; 3) document selection and appraisal; 4) data extraction; 5) data synthesis. The co-production group will help to map the current health and social care research system landscape, identifying key issues that may inhibit, or facilitate inclusion of people from BAFDC in health and social care research. Data will be extracted, analysed and synthesised following realist logic analysis, identifying and explaining how context and mechanisms are conceptualised in the literature and the types of contextual factors that exist and impact on inclusion and participation of people from BAFDC. Findings will be reported in accordance with Realist and Meta-narrative Evidence Synthesis Evolving Standards (RAMESES) guidance.

ETHICS AND DISSEMINATION

The co-production group will agree an ethical approach considering accountability, responsibility and power dynamics, by establishing a Terms of Reference, taking a reflexive approach and co-producing an ethical framework to guide the review. Findings will be disseminated to Black African Diaspora Communities and the research community, using a variety of communication methods including arts-based, through peer-reviewed publications and conference presentations, agreeing a strategy for dissemination with people from BAFDC, charities, organisations and key stakeholders with the co-production group.

STRENGTHS AND LIMITATIONS

To our knowledge, this will be the first rapid realist review undertaken exploring the contextual factors that influence inclusion and participation and identification of the mechanisms that facilitate inclusion and participation of people from BAFDC in health and social care research, internationally.

Co-producing the review with people from BAFDC with lived and professional experience, will facilitate a deeper understanding of current barriers and facilitators affecting the inclusion and participation of people from BAFDC in health and social care research, acting as content experts, drawing on their experiential knowledge which is rarely reported in formal literature.

This study will help to inform the development of intervention reflected in the methods, that enable greater participation of people from BAFDC in health and

For peer review only

social care research in the UK and may also be applicable to other health and social care research systems.

Keywords Under-representation, Health Inequities, Black People, Participation, Biomedical Research

For peer review only

INTRODUCTION

Research aims to enhance the health outcomes of the whole population [1, 2]. However, the lack of participation[1] in health and social care research studies by under-served groups may contribute to these groups not reaping the benefits of scientific discoveries [3-12]. One of the most affected underserved groups are Black and ethnic minority groups [13].

Studies suggest that Black and ethnic minority populations are most affected by health inequities and have historically been ignored, marginalised and forgotten in health and social care research [24,13,27]. In the UK, Black African Diaspora Communities (BAFDC) are a minoritised group that includes Black, Black British, Black Welsh, Caribbean or African, and individuals with dual/multiple heritage and other groups who have Black African lineage [25, 28, 29,14]. Minoritised groups are defined as having their "*cultural, political and social power*" destroyed because of their identity, by structures and processes that uphold power and domination [14, 15]. For the purposes of this review, people from BAFDC refers to adults aged ≥18 years, as children and youths from BAFDC, warrants a separate review. Little progress has been made in improving health outcomes for individuals from BAFDC [16-20].

In the UK, Black women are four times more likely to die during pregnancy, or up to one year after giving birth [16], diabetes prevalence is up to three times greater in BAFDC [17], one in four Black men will develop prostate cancer [18], and the

[1] Participation in health and social care research as defined by the UK's largest funder of research, the National Institute for Health and Care Research (NIHR), is when people take part in a research study [19]. This includes experiences of being recruited into a study, such as a clinical trial, or participating in focus groups, or completing a survey, for example [20].This differs from Patient and Public Involvement Engagement (PPIE) in health and social care research, where patients and members of the public are actively involved as partners in the research process across a range of activities in, such as commenting on research materials, or being part of a steering group or contributing to the synthesis of results [19].

For peer review only - http://bmjopen.bmj.com/site/about/guidelines.xhtml

prevalence of hypertension and stroke are also higher in this population [16-18, 21, 22].

Such avoidable and unwarranted differences in health, are the culmination of social, economic, environmental, and structural disparities caused by the unequal distribution of power and resources, underpinned by structural inequalities and racism [23, 24]. However, it remains unclear as to how genetic determinants may contribute to the higher incidence of certain diseases amongst people from BAFDC, primarily due to a lack of representation of these populations in genomics research [31].Enabling representation of people from BAFDC in health and social care research is critical for innovation and new discoveries; data science; ensuring access to specific therapeutic drugs, or treatments; building trust in science and medicine; addressing health disparities and ensuring provision of evidence-based healthcare [12, 25]. Without this, there is a risk of perpetuating health inequalities and social injustice with data producing algorithms used to guide treatment decisions based on White populations [25]. Furthermore, the potential benefits of personalised medicine and access to new treatments such as in cancer trials may not be realised if people from BAFDC are excluded from research [26]. Additionally, through improving the health and well-being of people from BAFDC, reducing health inequalities may provide economic benefits for the National Health Service (NHS), providing better value for taxpayers [27]. The responsibility to improve participation has often been directed at individuals who are under-represented. Terms such as “hard-to-reach”, have been frequently used to describe individuals who are marginalised, or disadvantaged, creating an unhelpful narrative that places the blame on individuals, as opposed to services being inaccessible due to a number of barriers such as studies not routinely reflecting the needs of under-served groups [28-30]. Health and

For peer review only

social care research is crucial for reducing health disparities and improving health and well-being globally, but without diverse participation and the representation of all groups, especially those who have some of the poorest health outcomes, social equity cannot be reached, creating an enduring challenge for some of the wealthiest, research-active countries [23, 31].

The science of inclusion

In comparison to the UK, the United States has had a lengthy history of policies implemented to improve diversity within health and social care research; consequently, the evidence base around inclusion and participation of Black and ethnic minority populations is largely from the US [12, 15, 32-39]. A report by The National Academies of Sciences Engineering and Medicine (NASEM) (2022) suggests that these policies have failed to reduce exclusion of under-served populations [12]. After the implementation of the *National Institute of Health (NIH) Revitalisation Act of 1993*, there was some reported increase in previously excluded groups, such as women, taking part in health and social care research, though individuals from Black and ethnic minority populations remained under-represented [38-41]. Guidance around improving inclusion is problematic when attempting to operationalise, reproduce or measure it [42, 43]. Adoption of NIH policies have not adequately encouraged researchers to widen their eligibility criteria, with the Federal Drugs Agency lacking the power to enforce recommendations, resulting in exclusion of the very groups the Act was developed [12]. Exclusion has been related to the lack of reporting of outcomes in research studies by race and ethnicity, impacting upon the evidence-base for clinical decision-making [12]. Exclusion is reported to have been observed in commercial cancer trials, where eligibility criteria can be very restrictive [44-46]. These trials often exclude individuals with comorbidities, which

For peer review only

are more prevalent in Black and ethnic minority populations, preventing access to new treatments, such as immunotherapy [45, 46]. The impact of eligibility criteria on inclusion requires further investigation to find solutions. Consequently, a move to develop a stronger body of knowledge about the science of inclusion is emerging, focussing on theory and empirical evidence related to the process of health and care research from initial engagement with individuals from Black and ethnic minority populations to participation in studies [12,26].

In the UK, the National Institute for Health and Care Research (NIHR) defines research inclusion as "*taking a whole systems approach to what we do and how we do it; identifying and removing longstanding, structural barriers to success across our people, policies, processes and practices*" [47].Robust evidence is needed to inform how the deficits in the infrastructure of health and social care research systems should be addressed in order to focus on inclusion and participation [48].There is a need to understand the contextual and causal factors that influence inclusion and participation and to develop the science of inclusion based on theory; clear explanations about theories of inclusion and participation of Black and ethnic minority populations are lacking [49].

The importance of inclusion

In the UK, there has been less focus on policy measures around inclusion in health and social care research (for example, there is a lack of practice and policy in regards to reporting of inclusion and assessments of quality of data collection), and more focus on recognising the importance of inclusion, and working to identify and address barriers to inclusion [12, 22, 47]. This contrast may reflect fundamental differences in the values and politics of the UK and US and the long history of race and ethnicity data collection policies and standards in the US [43, 50, 51]. The UK's

For peer review only

National Institute for Health and Care Research (NIHR) has acknowledged the importance of inclusion in health and social care research by developing and implementing a *Research Inclusion Strategy 2022-2027* demonstrating commitment to a long-term inclusivity agenda [43]. A focus on widening access and participation for greater diversity and inclusion is a critical aspect of the strategy, indicating that "*work is required to initiate more inclusive recruitment strategies that promote participation from under-served groups nationwide*" [43]. Creating a more equitable health and social care research system is crucial to ensuring disadvantaged groups most affected by health inequities benefit from participating in research and feel that research is relevant to them [25].

Lack of progress

Globally, little progress has been made on participation of Black populations in research studies [12]. There is strong evidence in the US to suggest that African Americans are willing to participate in research, and the decision to participate may be influenced by mechanisms situated at other levels of the health and social care research system (such as the intrapersonal and structural levels, rather than at an individual level) [12, 43]. There are reports that willingness to participate has been misrepresented due to biases held by researchers, though the mechanisms that cause bias to function at the intrapersonal and structural levels of health and social care systems are poorly understood [12, 43]. It has been suggested that the responsibility for developing interventions that will improve inclusion and participation of people from BAFDC lies with researchers, institutions, decision-makers, policymakers and funders of research [12, 52]. Such interventions are required at multiple levels of the health and social care research system including policymaking and funding to provide resources to a specific population with the aim of creating

For peer review only

progressive change [39]. The way in which an intervention is developed and implemented is dependent upon the power of individuals and institutions and how they manage its implementation, which is pertinent when considering the architecture required for an intervention, for a marginalised population such as BAFDC [53]. For example, if an intervention is designed and implemented by individuals from a perceived dominant population, it may lack the perspectives of a diverse population which may impact upon the success of its design [13]. For an intervention to have the best chance of success, it is crucial to understand the context within which it will be implemented, as well as the mechanisms by which it works. Consequently, a realist approach provides the most suitable method for developing new thinking through offering a deeper, more pragmatic explanation than a systematic review [54]. While systematic reviews offer an assessment of the effectiveness of interventions, they often lack explanations as to how and why an intervention may, or may not have worked, which is particularly valuable in complex social systems, such as the health and social care research system [50]. The learning from this realist review will be a timely addition to existing efforts. We are at a pivotal point in addressing the structural, racial injustices that have been brought to the fore by critical events over recent years, such as the murder of George Floyd and the COVID-19 pandemic that disproportionately impacted Black and ethnic minority communities [24, 55, 56].

METHODS AND ANALYSIS

Our positionality statement can be found in online supplemental file 1.

Aims

This rapid realist review will broadly explore secondary data to provide insights that will contribute to the development of a realist theory of inclusion and participation in

For peer review only

health and social care research by people from BAFDC in the UK. The theory may be applicable to other health and social care research systems internationally. It will be undertaken with a co-production group composed of people from BAFDC who have the lived experience of the challenges being investigated [57]. A rapid realist review benefits from “content experts” such as the co-production group, improving the efficiency of the review through helping to focus the scope of the review, development of the initial programme theory and refining the search process and accurate interpretation of the results [58]. Rapid realist reviews are reliable tools for policymakers who require evidence for urgent policy issues [58].The review will help to explain the context of the health and social care research system, identifying underlying causal factors that influence inclusion and participation of people from BAFDC in health and social care research. We will develop a programme theory through following five key steps as recommended by Pawson et al (2005) that explains how inclusion and participation may be facilitated with people from BAFDC in health and social care research [59, 60]. This programme theory is intended to inform the development of an intervention to improve inclusion and ultimately outcomes for people from BAFDC.

Review questions

1. What are the contextual and/or causal factors that influence inclusion and participation in health and social care research by people from BAFDC?
2. How and where do mechanisms occur that underly implicit and complicit bias that affects the inclusion and participation of people from BAFDC in health and social care research?
3. What barriers and facilitators do people from BAFDC feel affects their experiences of participation in health and social care research?

For peer review only

4. What might the components of an intervention(s) look like that fosters inclusion and participation of people from BAFDC in health and social care research?

Study design

The design will be guided by Pawson et al.'s five steps and in accordance with RAMESES for quality and reporting [61]. Figure 1 shows a diagram of the review process as adapted from Duddy and Wong [62]. Due to the complexity of context, the focus of the review will be steered by a unique theoretical framework developed with the co-production group from pre-existing mid-range theories deemed pertinent to this research. Theories will be identified through analysis of resources from the review team, as well as comprehensive evaluation of the literature [55]. A realist review is a theory-driven method for investigating complex interventions, or programmes [54]. Evidence is produced in the form of theories that are best understood by analysing the contexts, mechanisms and outcomes of a programme [54]. Understanding context can help to create an optimal condition for the successful implementation of a programme or intervention [63]. The interaction between context (C) and mechanism (M) results in an outcome (O) [54]. This formula is known as a CMO configuration (CMOC) and is a way of explaining theory in realist research [54].

INSERT Figure 1

Stakeholder involvement

Stakeholder involvement is key to realist research and will be achieved by using a co-production approach [64]. Stakeholders are people who may have an interest in the research being carried out, may be involved in shaping it and could be affected by the outcomes [65]. Co-production is a way of sharing power, taking into account

For peer review only

the skills and many perspectives within a group of people - it involves respecting and valuing the knowledge of others as well as providing reciprocity and focussing on building and maintaining relationships [66].

Recruitment

A co-production group of eight individuals (VE, TK, DG, LH, EM, TO, IS, SW) from BAFDC will meet to share knowledge and identify training needs, around the realist approach, prior to the review. Individuals were approached by the lead author (EH) through her existing networks (some had already been involved in Patient Public Involvement during the development phase of the research proposal): directly approaching Black community organisations and individuals, providing written information about co-production and the research project, with 1:1 meetings held virtually with all who were interested, to provide an opportunity to ask questions and meet EH. The co-production group includes Experts by Experience, as well as stakeholders from the health and social care research system who self-identify as belonging to BAFDC. In this review, Experts by Experience are regarded as patients and members of the public with lived experience of the health and social care research system [67].

Work schedule

We will map out the review process and draw on expertise from within the group, through creative methods and discussion, to understand how the health and social care research system currently serves people from BAFDC in the UK. The co-production group will be consulted in alignment with the principles of co-production throughout, as key “experts” with additional stakeholders sought for expertise, if required [66]. The group will be consulted throughout the review via virtual meetings on Microsoft Teams and email, allowing for wider geographic inclusion as the group

For peer review only

is based in the West Midlands and London regions, improving accessibility, with sessions kept to a maximum of 1½ hours to avoid digital fatigue [68].There will also be at least one face-to-face meeting, held at a central location convenient to the group, to allow for longer discussion and to help build relationships.

Sequence of steps

The review will be conducted following five steps: 1) shaping the scope of the review 2) searching for the evidence 3) document selection and appraisal, which will be based on relevance and rigor 4) data extraction and organisation of evidence will be conducted iteratively 5) data synthesis will be used to explain deficits in context and how they impact on inclusion and participation and development of programme theory.

Step 1: Shaping the scope of the review: key concepts and construction of a theoretical framework

Programme theories create a scaffolding to explore how, why, for whom and under what circumstances complex programmes, or interventions work [69].In realist terms, an intervention is a theory [70].This stage will focus on the development of a theoretical framework that will shape the focus of the review, support data extraction, appraisal and synthesis, and identify concepts around inclusion and participation of people from BAFDC in health and social care research [70]. Evidence suggests that a small number of theories can be developed into a framework that will support the development of initial programme theory that incorporates the multiple levels of a system [70]. To identify relevant theories, concepts critical to inclusion and participation of people from BAFDC will be identified through creative discussion with the co-production group using Lego Serious Play (LSP) as a method for facilitating their input [71]. A small pilot with NIHR Research Champions, or members of the

For peer review only

public who have experience of being involved and engaged in health and social care research will take place prior to using the approach with the co-production group.

Outputs

We will produce a simple, research participant pathway representing the health and social care research process that will be used as an artifact, to consult with the co-production group to stimulate discussion during LSP, to support the facilitation of individual's thoughts and reasoning [72] (see Figure 2).

INSERT Figure 2

The co-production group will use LSP to map the existing health and social care research system. Concepts from other programmes in related areas can also be drawn upon. Existing mid-range theories will then be identified through an initial scoping search based upon key concepts that arise following the LSP activity. A list of these mid-range theories will be presented to the co-production group to decide whether they should be included in the theoretical framework, based upon their importance to the research aim and relevance to people from BAFDC. Theories deemed pertinent by the group will be merged into a framework to guide data extraction and appraisal. Evidence will be collected to theorise how an intervention may work, facilitating identification of CMOC's through matching to existing theories, while considering information that may be missing from existing mid-range theories [54, 73].

Step 2: Searching for evidence

A search for evidence will be conducted, including international literature, collecting a wide range of secondary data sources and grey literature including: all types of studies, peer-reviewed articles, opinion pieces, commentaries, conference papers/proceedings, reports, and social media excerpts [61]. In discussion with an

For peer review only

Information Specialist following a pilot search of the evidence it was felt that no time limit should be applied to the initial search due to a low return of papers and to ensure that the history of research previously conducted addressing the issue of inclusion and participation in health and social care research is captured. We expect further adjustments to the search strategy to be required. Discussions and decision-making with the co-production group will be held around relevance of international literature to a UK context. A data extraction form will be informed by the theoretical framework as a way of combing the evidence and appraising it through scoring it as high, moderate, low based upon its usefulness and relevance [74].

Data sources and search strategy

An initial search will be developed based on the theoretical framework, with key search terms identified in Table 1.

INSERT Table 1

Using this strategy, a search for the evidence will be conducted using the following databases: Medline, EMBASE, PsycINFO, Web of Science, Race Relations Abstracts, Sociological Abstracts, University of the West Indies, and Patient-Centred Outcomes Research Institute (PCORI). A search of grey literature will be conducted (Table 2), although recommendations from the co-production group may also evolve as the review progresses. Literature sources will be prioritised by the co-production group where relevant.

INSERT Table 2

Screening of abstracts will be carried out according to inclusion and exclusion criteria developed, based upon the theoretical framework, with no date limit initially. This will be reviewed following the database search and a limit set, should the return of documents be excessive.

Screening process

EH will undertake screening based on title and abstract and a second reviewer will screen a random sample of 10% of the identified citations, setting a kappa measure of k> 0.8 to measure inter-rater reliability [61]. This will be calculated using the formula for Cohen's kappa [75]. Reviewers will discuss disagreements with the co-production group and research team. If k<0.8 a further 10% sample will be screened, and the outcome discussed with the research team. Should an abstract not be available (for example, in some grey literature), documents will be included and full screening of each will be carried out at second level screening. *Rayyan* referencing software will be utilised for screening as it enables blinding of screening decisions, collaboration functions and presentation of screening decisions in a simple format which can be exported onto a spreadsheet [76]. Papers that are excluded by one reviewer and included by another will be brought to the wider research team for further discussion, as well as those where one, or both reviewers are unsure.

Iterative searching

As realist reviews are iterative, searching for additional data to support theory development is likely [54]. For each additional search, the research team will develop further inclusion/exclusion criteria, with each search tested and refined through support from an Information Specialist and screening conducted as summarised above.

Step 3: selection and appraisal

After initial screening in step 2, documents will be selected based upon relevance and rigour after the full text has been read by EH and inclusion/exclusion criteria applied [77] (see Table 3). Relevant documents are those which are deemed helpful and valuable for building theory whilst rigour ascertains the trustworthiness and

For peer review only

credibility of methods used to produce data [77]. Although, methodological quality of papers is not so important in realist research, relevance and robustness is [78]. Evidence may include a wide range of sources e.g. opinion pieces that may capture important evidence based upon experiences [79]. Pawson asserts that even poor evidence, which may be methodologically weak, can provide rich insights and thus following a hierarchical approach to selecting documents should not pertain to realist reviews [77, 80, 81]. Realist research is informed by evidence, as opposed to evidence-base and therefore a diverse range of literature sources are needed to develop programme theories with sources selected according to relevance and rigour [81].

INSERT Table 3.

Step 4: extracting and organising data

Databases will be developed to extract and organise data, tailored for this review. Data will be extracted by EH with a 10% subsample of included papers, also completed by the second reviewer. Disagreements will be discussed with the research team and co-production group. If kappa is <0.8 then a further sub-sample of 10% will be reviewed by the second reviewer. We will pilot the use of *NVivo* software as an alternative to *Excel* to assist data coding and organisation; we will code three to five documents in both databases to determine which is the most pragmatic tool for this review [82]. Extracted data will be centred on dialogue that helps to build programme theory [83]. Codes will be developed both inductively from documents, and deductively from the theoretical framework [84]. Data will be organised into broad, conceptual categories , through grouping the codes together and prioritising coding for causal insight in regards to the architecture of the health

For peer review only

and care research system [83]. This will support identifying patterns in the data and configuring data into CMOCs[78] .

Step 5: analysis and synthesis

Data analysis and synthesis will be led by EH with reflections and discussion amongst the research team and co-production group to support interpretation of CMOC's using retroduction [85]. Retroduction allows the researcher to incorporate intuitions and insights, as well as inductive and deductive logic, considering the causal forces that may be producing what is observed [85]. This theorising is based upon the researchers' interpretations and may not be testable in the first instance, particularly if only part of a theory is developed [85]. The co-production group will be involved in co-producing this, to ensure that the developing programme theory, is true to their lived and professional experiences. The group will decide meeting frequency, location, and length.

Strengths and limitations

A key strength of this realist review lies with the co-production group that will be involved throughout [59]. The combined skills and expertise of a group of stakeholders from BAFDC, with both lived and professional experience in the health and social care research system will place people from BAFDC at the centre of this review. The group's lived experience of being Black, or having a shared history of "*colonialism and enslavement in the past and continuing to experience racism and diminished opportunities*" makes them experts in their own right and will ensure that their reality cannot be denied [86]. A limitation is that the lead researcher (EH) is White British and has not experienced racism. Therefore, she will reflect on her positionality throughout, with a willingness to engage, learn, and take on board the complex issues that may present. The co-production group feel that Tuckman's

For peer review only

model of "*forming, storming, norming and performing*" is a useful pathway for reflecting on how the group may develop to ensure the aims and objectives of this project are met and to create a progressive group culture [87]. This review will be undertaken as a rapid realist review, in order to answer timely research questions for which there is limited published evidence on interventions or programmes that effectively foster inclusion and participation in health and social care research with people from BAFDC [58, 88]. Limitations may include not being able to access or include all sources identified in searches. However, together as a research team and co-production group, we will agree on the priorities of the programme theory with a view to conducting future research on additional aspects that we are unable to address in this project.

ETHICS AND DISSEMINATION

This review does not require ethical approval due to the use of secondary data and Patient Public Involvement (PPI) in the form of co-production. The review will be registered with PROSPERO. However, ethical considerations around accountability, responsibility and power dynamics will be discussed and reflected upon, with the co-production group and research team. This review will lead to a programme theory that begins to explain what may work, for supporting inclusion and participation of people from BAFDC in health and social care research internationally. This theory will continue to be built through qualitative data collection methods, with stakeholders from each level of the health and social care research system, in a realist evaluation. Further outputs will include a set of principles, recommendations and a conceptual framework that will inform the development of an intervention suitable for implementation, to support a UK research system focussed on equity and social justice [12]. An early prototype for an intervention, based on the findings, will be

For peer review only

developed with the co-production group. Outputs for people from BAFDC will be co-produced and will focus on a series of community engagement events, dissemination of findings via radio, in newspapers such as *The Voice*, a well-established British African-Caribbean newspaper since 1982 and via social media.

CONCLUSION

This realist review has the potential to explain how an intervention for facilitating inclusion and participation in the health and social care research system in the UK with people from BAFDC should work [89]. This may also be useful to international health and social care research systems. As described by the co-production group, it will centre and amplify the voices of people from BAFDC through co-production, ensuring the "voiceless" are heard and not ignored, advancing understanding of the complexity of participation as well as building trust and new relationships with people from BAFDC. Centring people from BAFDC through co-production in this project, will require reflexivity by the researcher, research team and co-production group, about how the involvement of people from BAFDC may be experienced. In addition, promoting reciprocity through identifying opportunities (e.g. co-authorship of peer-reviewed papers), that benefit the co-production group and nurtures respect between the researcher and the group will be actively pursued [90]. Co-production relies on the development of relationships and the involvement of people from BAFDC will influence what is discussed and how this project is shaped with acknowledgement of privilege and possible power imbalances continually reflected upon [91, 92]. Marginalised and under-served groups must be placed at the centre of how we design an inclusive health and social care research system, to ensure equity and social justice [29,38].

Figure 1. Realist review project plan

For peer review only

Figure 2. Pathway of experience of participation

Table 1. Search terms

Table 2. Grey literature sources

Table 3. Inclusion Exclusion criteria

Authors' contributions EH conceived the study with input from SS, DE, JD and RS. EH wrote the first draft of this manuscript and all co-authors provided criticism and refinement. All authors approved the final version.

Funding statement EH is funded by the NIHR [Health Education England/National Institute for Health and Social Care Research Clinical Doctoral Research Fellowships programme, grant number 302121]. SS is part funded by the NIHR Applied Research Collaboration (ARC) West Midlands, the NIHR Health Protection Research Unit (HPRU) Gastrointestinal Infections, the NIHR HPRU Genomics and Enabling data, the NIHR West Midlands Evidence Synthesis Group and the NIHR Health Determinants Research Collaboration Coventry.

Competing interests There are no competing interests.

References

1. Gov.uk. Saving and Improving Lives: The Future of UK Clinical Research Delivery [online].2021. https://www.gov.uk/government/publications/the-future-of-uk-clinical-research-delivery/saving-and-improving-lives-the-future-of-uk-clinical-research-delivery (accessed 18 December 2022)
2. WHO. WHO's goal : Forward looking and prioritised global health research [online].2023. https://www.who.int/our-work/science-division/research-for-health (accessed 3 April 2023).
3. Iyizoba-Ebozue Z, et al Reflection on Black and Ethnic Minority Participation in Clinical Trials. Clinical Oncology (Royal College of Radiologists), 2022; 34(10):674-677. doi:10.1016/j.clon.2022.07.006
4. Smart A and Harrison E The under-representation of minority ethnic groups in UK medical research. Ethn Health, 2019;22(1): 65-82. doi: 10.1080/13557858.2016.1182126

For peer review only

5. Treweek S, et al COVID-19 and ethnicity: who will research results apply to? The Lancet, 2020; 95(10242):1955-1957. doi:10.1016/S0140-6736(20)31380-5
6. Sheppard V, et al Recruitment of African Americans into Cancer Clinical Research: Strategies and Outcomes. Journal of Urban Health, 2021;98(Suppl 2):149-154. doi:10.1007/s11524-021-00565-8
7. Lau Y et al Does Ethnicity Influence Recruitment into Clinical Trials of Parkinson's Disease? Journal of Parkinsons Disease, 2022;12(3):975-981. doi:10.3233/JPD-213113
8. Iqbal H et al A systematic review to identify research priority setting in Black and minority ethnic health and evaluate their processes. PLoS ONE [Electronic Resource], 2021. 16(5): p. e0251685.
9. Cunningham-Erves J et al A pilot study of a culturally-appropriate, educational intervention to increase participation in cancer clinical trials among African Americans and Latinos. Cancer Causes & Control, 2021; 32(9):953-963. doi:10.1007/s10552-021-01449-7
10. De las Nueces D et al A systematic review of community-based participatory research to enhance clinical trials in racial and ethnic minority groups. Health Services Research 2012 47(3 Pt 2):1363-1386. doi:10.1111/j.1475-6773.2012.01386.x;
11. McFarlane S, et al Community-Based Participatory Research (CBPR) to Enhance Participation of Racial/Ethnic Minorities in Clinical Trials: A 10-Year Systematic Review, Health Communication.2022; 37(9):1075-1092. doi:10.1080/10410236.2021.1943978
12. National Academies of Sciences, Engineering, and Medicine (NASEM) Improving Representation in Clinical Trials and Research : Building Research Equity for Women and Underreprsented Groups, ed. K.B.-D.a.A. Helman. Washington DC: National Academies Press 2022
13. Williams D et al Racism and Health: Evidence and Needed Research. Annu Rev Public Health, 2019; 40:105-125. doi:10.1146/annurev-publhealth-040218-043750
14. Selvarajah S, et al Categorisation and Minoritisation. BMJ Global Health, 2020; 5(12):e004508. doi:10.1136/bmjgh-2020-004508
15. Awidi M and S Hadidi Participation of Black Americans in Cancer Clinical Trials: Current Challenges and Proposed Solutions. JCO Oncology Practice, 2021;17(5):265-271. doi:10.1200/OP.21.00001
16. MBRRACE-UK. MBRRACE-UK Perinatal Mortality Surveillance: UK Perinatal Deaths for Births from January to December [online] 2021 https://www.npeu.ox.ac.uk/mbrrace-uk/reports/perinatal-mortality-surveillance (accessed 2 Feb 2023)
17. Bello O, et al Black African men with early type 2 diabetes have similar muscle, liver and adipose tissue insulin sensitivity to white European men despite lower visceral fat. Diabetologia, 2019; 62(5):835-844. doi:10.1007/s00125-019-4820-6

18. Prostate Cancer UK. Prostate cancer and other prostate problems: Information for black men [online] 2023 https://shop.prostatecanceruk.org//our-publications/all-publications/prostate-cancer-and-other-prostate-problems-information-for-black-men?limit=100 (accessed 8 Jul 2023)
19. NIHR. Glossary [online] 2023 https://www.nihr.ac.uk/about-us/glossary.htm?letter=P&postcategory=-1 (accessed 11 May 2023)
20. NIHR. Briefing notes for researchers - public involvement in NHS, health and social care research [online] 2021 https://www.nihr.ac.uk/documents/briefing-notes-for-researchers-public-involvement-in-nhs-health-and-social-care-research/27371 (accessed 28 Jan 2023)
21. The King's Fund.The health of people from ethnic minority groups in England [online] 2021 https://www.kingsfund.org.uk/publications/health-people-ethnic-minority-groups-england (accessed 20 Apr 2023)
22. The King's Fund.Tackling the worst health outcomes: a 2020-24 strategic priority [online] 2020 https://www.kingsfund.org.uk/priorities/tackling-worst-health-outcomes (accessed 20 Apr 2023)
23. Rai T, et al What would it take to meaningfully attend to ethnicity and race in health research? Learning from a trial intervention development study. Sociology of Health & Illness, 2022; 44 Suppl 1(Suppl 1):57-72. doi:10.1111/1467-9566.13431
24. Nobles M, et al RACISM Overcoming science's toxic legacy. A Nature special issue Nature [online] 2022 https://www.nature.com/immersive/d42859-022-00031-8/index.html (accessed 1 Nov 2022)
25. Knight H, et al Challenging racism in the use of health data. The Lancet Digital Health, 2021; 3(3):e144-e146. doi:10.1016/S2589-7500(21)00019-4
26. The Lancet. Personalised medicine in the UK.2017; 391(10115): e1 doi:https://doi.org/10.1016/S0140-6736(17)33261-0
27. Lewis T and Wenzel L, Equity and endurance: how can we tackle health inequalities this time? [online] 2022 https://www.kingsfund.org.uk/publications/how-can-we-tackle-health-inequalities (accessed 28 Mar 2023)
28. NSPCC. Why language matters: reframing responsibility for accessing services [online] 2022 https://learning.nspcc.org.uk/news/why-language-matters/not-hard-to-reach-reframing-responsibility-for-accessing-services (accessed 15 Apr 2023)
29. Flanagan S and Hancock B 'Reaching the hard to reach' - lessons learned from the VCS (voluntary and community Sector). A qualitative study. BMC Health Services Research, 2010; 10:92. Published 2010 Apr 8. doi:10.1186/1472-6963-10-92
30. Witham M, et al Developing a roadmap to improve trial delivery for under-served groups: results from a UK multi-stakeholder process. Trials, 2020; 21(1):694. Published 2020 Aug 1. doi:10.1186/s13063-020-04613-7

For peer review only

31. Sharma A, et al Patient and caregiver attitudes towards gene therapy for sickle cell disease: A need for partnership and education. Blood, 2021; 138 (Supplement 1): 918. doi: https://doi.org/10.1182/blood-2021-148795

32. Rosen C, et al Diversity in Clinical Trials — Next Steps. New England Journal of Medicine, 2022. 387(15) e34 doi:10.1056/nejmp2201379

33. Dawson S, et al Black and minority ethnic group involvement in health and social care research: A systematic review. Health Expectations, 2018; 21(1):3-22. doi:10.1111/hex.12597

34. George S, et al A systematic review of barriers and facilitators to minority research participation among African Americans, Latinos, Asian Americans, and Pacific Islanders. Am J Public Health, 2014;104(2):e16-e31. doi:10.2105/AJPH.2013.301706

35. Camidge D et al Race and ethnicity representation in clinical trials: findings from a literature review of Phase I oncology trials. Future Oncology, 2021; 3271-3280. doi:10.2217/fon-2020-1262

36. Kwiatkowski K, et al Inclusion of minorities and women in cancer clinical trials, a decade later: Have we improved? Cancer, 2013; 119(16):2956-2963. doi:10.1002/cncr.28168

37. Rodríguez-Torres E, et al Barriers and facilitators to the participation of subjects in clinical trials: An overview of reviews. Contemp Clin Trials Commun, 2021; 23:100829. Published 2021 Aug 3. doi:10.1016/j.conctc.2021.100829

38. NIH National Institutes of Health Revitatlization Act of 1993[online] 1993 https://orwh.od.nih.gov/sites/orwh/files/docs/NIH-Revitalization-Act-1993.pdf (accessed 10 Dec 2022)

39. Mays V Research challenges and bioethics responsibilities in the aftermath of the presidential apology to the survivors of the U. S. Public Health Services Syphilis Study at Tuskegee. Ethics & Behavior, 2012; 22(6):419-430. doi:10.1080/10508422.2012.730787

40. Meinert J, et al Bridging the gap: Recruitment of African-American women into mental health research studies. Academic Psychiatry, 2003; 27(1):21-28. doi:10.1176/appi.ap.27.1.21

41. Topp R, et al Including African Americans in health care research. Western Journal of Nursing Research, 2008; 30(2):197-203. doi:10.1177/0193945907303063

42. NIHHealth Report of the Advisory Committee on Research on Women's Health: Fiscal Years 2017–2018 [online] 2019 https://orwh.od.nih.gov/our-work/fiscal-years-2019-2020-full-report-on-research-on-womens-health (accessed 10 Jan 2022)

43. Gilmore-Bykovskyi A, et al Traversing the Aging Research and Health Equity Divide: Toward Intersectional Frameworks of Research Justice and Participation. Gerontologist, 2022; 62(5):711-720. doi:10.1093/geront/gnab107

For peer review only

44. Reihl S, et al A Population Study of Clinical Trial Accrual for Women and Minorities in Neuro-Oncology Following the NIH Revitalization Act. medRxiv, 2021; 24(8):1341-1349. doi:10.1093/neuonc/noac011
45. Nazha B, et al Enrollment of Racial Minorities in Clinical Trials: Old Problem Assumes New Urgency in the Age of Immunotherapy. American Society of Clinical Oncology Educational Book, 2019; 39:3-10. doi:10.1200/EDBK_100021
46. Jiagge E, et al Participatory Action for Access to Clinical Trials (PAACT): Increasing participation of blacks in Southeastern Michigan in cancer clinical trails. Journal of Clinical Oncology 2022; 40(16): e18527-e18527 doi: 10.1200/JCO.2022.40.
47. NIHR Equality, Diversity and Inclusion Strategy 2022-2027 [online] 2022 https://www.nihr.ac.uk/documents/equality-diversity-and-inclusion-strategy-2022-2027/31295 (accessed 28 Nov 2022)
48. Jackson J The Challenge of Health Equity in Clinical Research Towards a Quantifiable Science of Inclusion [online] 2020 https://www.nia.nih.gov/research/alzheimers-dementia-outreach-recruitment-engagement-resources/challenge-health-equity (accessed 12 Jan 2023)
49. Archives.gov Civil Rights Act 1964 [online] 1964 https://www.archives.gov/milestone-documents/civil-rights-act (accessed 28 Oct 2022)
50. Ham C Money can't buy you satisfaction. Bmj, 2005; 330(7491):597-599. doi:10.1136/bmj.330.7491.597
51. Díaz Rios L, et al Collecting, Using, and Reporting Race and Ethnicity Information: Implications for Research in Nutrition Education, Practice, and Policy to Promote Health Equity. Journal of Nutrition Education and Behavior, 2022;54(6):582-593. doi:10.1016/j.jneb.2022.01.006
52. National Academies of Sciences, Engineering, and Medicine (NASEM); Health and Medicine Division; Board on Population Health and Public Health Practice; Baciu A, et al eds. Communities in Action: Pathways to Health Equity. Washington (DC): National Academies Press (US) 2017 doi: 10.17226/24624
53. von Thiele Schwarz U, et al How to design, implement and evaluate organizational interventions for maximum impact: the Sigtuna Principles. European Journal of Work and Organizational Psychology, 2021; 30(3):415-427. Published 2020 Aug 26. doi:10.1080/1359432X.2020.1803960
54. Pawson R, et al Realist review--a new method of systematic review designed for complex policy interventions. J Health Serv Res Policy, 2005; 10 Suppl 1:21-34. doi:10.1258/1355819054308530
55. Hearst M, et al COVID-19 and Systemic Racism Pandemics Impact Daily Life for Immigrant/Refugees, Minneapolis, USA. Ann Glob Health, 2021; 87(1):107. Published 2021 Nov 9. doi:10.5334/aogh.3411

For peer review only

56. Delan Devakumar I, et al The Lancet Series on racism, xenophobia, discrimination, and health. The Lancet 2022; 395(10242):e112-e113. doi:10.1016/S0140-6736(20)31371-4

57. Griffiths S, et al Engaging Stakeholders in Realist Programme Theory Building: Insights from the Prospective Phase of a Primary Care Dementia Support Study. International Journal of Qualitative Methods, 2022; 21:16094069221077521 doi.org/10.1177/16094069221077521

58. Saul J et al A time-responsive tool for informing policy making: rapid realist review. Implementation Science, 2013 ;8:103. Published 2013 Sep 5. doi:10.1186/1748-5908-8-103

59. Pawson R and Tilley N Realistic Evaluation. London: Sage Publications 1997

60. Pawson R, et al Realist review-a new method of systematic review designed for complex policy interventions. Journal of health services research & policy, 2005; 10 Suppl 1:21-34. doi:10.1258/1355819054308530

61. Wong G, et al RAMESES publication standards: realist syntheses. BMC Med., 2013 ;11:21. Published 2013 Jan 29. doi:10.1186/1741-7015-11-21

62. Duddy C and Wong G Explaining variations in test ordering in primary care: protocol for a realist review. B. BMJ Open, 2018; ;8(9):e023117. Published 2018 Sep 12. doi:10.1136/bmjopen-2018-023117

63. Greenhalgh J and Manzano A Understanding ‘context’ in realist evaluation and synthesis. International Journal of Social Research Methodology, 2022; 25:5, 583-595, doi: 10.1080/13645579.2021.1918484

64. Emmel N et al.Doing Realist Research. London: Sage Publications .2018

65. Vitae. Research Project Stakeholders [online] 2022 https://www.vitae.ac.uk/doing-research/leadership-development-for-principal-investigators-pis/leading-a-research-project/applying-for-research-funding/research-project-stakeholders (accessed 12 Aug 2023)

66. NIHR Guidance on co-producing a research project [online] 2021 https://www.learningforinvolvement.org.uk/content/resource/nihr-guidance-on-co-producing-a-research-project/ (accessed 15 Oct 2022)

67. NIHR Policy Research Unit Older People and Frailty. Experts by Experience [onlin] 2023 https://www.opfpru.nihr.ac.uk/our-people/experts-by-experience/#:~:text=We%20consider%20experts%20by%20experience,patients (accessed 12 Jun 2023)

68. Williams O, et al COVID-19 and Co-production in Health and Social Care Vol 2: Volume 2: Co-production Methods and Working Together at a Distance. Policy Press [online] https://research-portal.uea.ac.uk/en/publications/covid-19-and-co-production-in-health-and-social-care-vol-2-volume (accessed 28 May 2023)

69. Flynn R, et al Developing an Initial Program Theory to Explain How Patient-Reported Outcomes Are Used in Health Care Settings: Methodological Process and Lessons Learned. International Journal of Qualitative Methods, 2020; 15(1):21-38. doi:10.1007/s40271-021-00530-2

70. Rycroft-Malone J, et al Realist synthesis: illustrating the method for implementation research. Implementation Science, 2012; 7:33. Published 2012 Apr 19. doi:10.1186/1748-5908-7-33
71. Garden C Lego Serious Play: Building engagement with cell biology. Biochem Mol Biol Educ, 2022; 50(2):216-228. doi:10.1002/bmb.21608
72. Nielsen M Using artifacts in brainstorming sessions to secure participation and decouple sequentiality. Discourse Studies, 2012. 14(1): 87-109 doi.org/10.1177/1461445611427211
73. Wong G, et al Internet-Based medical education : a realist review of what works, for whom and in what circumstances. BMC Med Educ, 2010; 10:12. Published 2010 Feb 2. doi:10.1186/1472-6920-10-12
74. Jagosh J CARES Realist Methodology Training [online]. 2022 https://realistmethodology-cares.org/dec2023realistsynthesismasterclass (accessed 10 Dec 2022)
75. Centre for Reviews and Dissemination CRD's guidance for u ndertaking reviews in health care [online] 2009 https://www.york.ac.uk/media/crd/Systematic_Reviews.pdf (accessed 10 Jan 2022)
76. Rayyan. Faster Systematic Reviews [online] 2022 https://www.rayyan.ai/ (accessed 15 Dec 2022)
77. Pawson R Evidence-based Policy: A Realist Perspective.California: Sage. 2006
78. Jagosh J, et al Critical reflections on realist review: insights from customizing the methodology to the needs of participatory research assessment. Research Synthesis Methods, 2014; 5(2):131-141. doi:10.1002/jrsm.1099
79. Wong G, et al RAMESES II reporting standards for realist evaluations. BMC Med Educ, 2016; 14(1):96. Published 2016 Jun 24. doi:10.1186/s12916-016-0643-1
80. Pawson R The Science of Evaluation: A Realist Manifesto. 2013, Los Angeles, California, London, England: Sage 2013;79(3), 580-582 doi.org/10.1177/0020852313497455
81. Hunter R, et al Realist review. International Review of Sport and Exercise Psychology, 2022; 15:1, 242-265, doi: 10.1080/1750984X.2021.1969674
82. Dalkin S, et al Using computer assisted qualitative data analysis software (CAQDAS; NVivo) to assist in the complex process of realist theory generation, refinement and testing. International Journal of Social Research Methodology, 2021; 24:1, 123-134,doi: 10.1080/13645579.2020.1803528
83. Bender J, et al Establishing best practices in cancer online support groups: protocol for a realist review. BMJ Open, 2021; 11(11):e053916. Published 2021 Nov 3. doi:10.1136/bmjopen-2021-053916
84. Gilmore B, et al Data Analysis and Synthesis Within a Realist Evaluation: Toward More Transparent Methodological Approaches. International Journal of Qualitative Methods, 2019; 18: doi.org/10.1177/1609406919859754

For peer review only

85. Greenhalgh T, et al Retroduction in realist evaluation. The RAMESES II Project [online] 2017 http://www.ramesesproject.org/media/RAMESES_II_Retroduction.pdf (accessed 2 Jun 2022)
86. UNISON. Defining Black [online] 2023 https://www.unison.org.uk/about/what-we-do/fairness-equality/black-members/defining-black/ (accessed 26 Jun 2023)
87. Tuckman B Developmental sequence in small groups. Psychological Bulletin, 1965; 63(6), 384–399 doi.org/10.1037/h0022100
88. Pawson R, et al Realist review--a new method of systematic review designed for complex policy interventions. J Health Serv Res Policy, 2005: p. 21-34.
89. Jagosh J, et al Benefits of realist evaluation for rapidly changing health service delivery. BMJ Open, 2022; 10 Suppl 1:21-34. doi:10.1258/1355819054308530
90. Staniszewska S, et al Co-production: a kind revolution. Res Involv Engagem, 2022; 8(1):4. Published 2022 Feb 5. doi:10.1186/s40900-022-00340-2
91. Pettican A, et al Doing together: reflections on facilitating the co-production of participatory action research with marginalised populations. Qualitative Research in Sport, Exercise and Health, 2022; 15:2, 202-219, doi: 10.1080/2159676X.2022.2146164
92. Farr M, et al Co-producing knowledge in health and social care research: reflections on the challenges and ways to enable more equal relationships. Humanities and Social Sciences Communications, 2021; 8(1): 105 doi.org/10.1057/s41599-021-00782-1

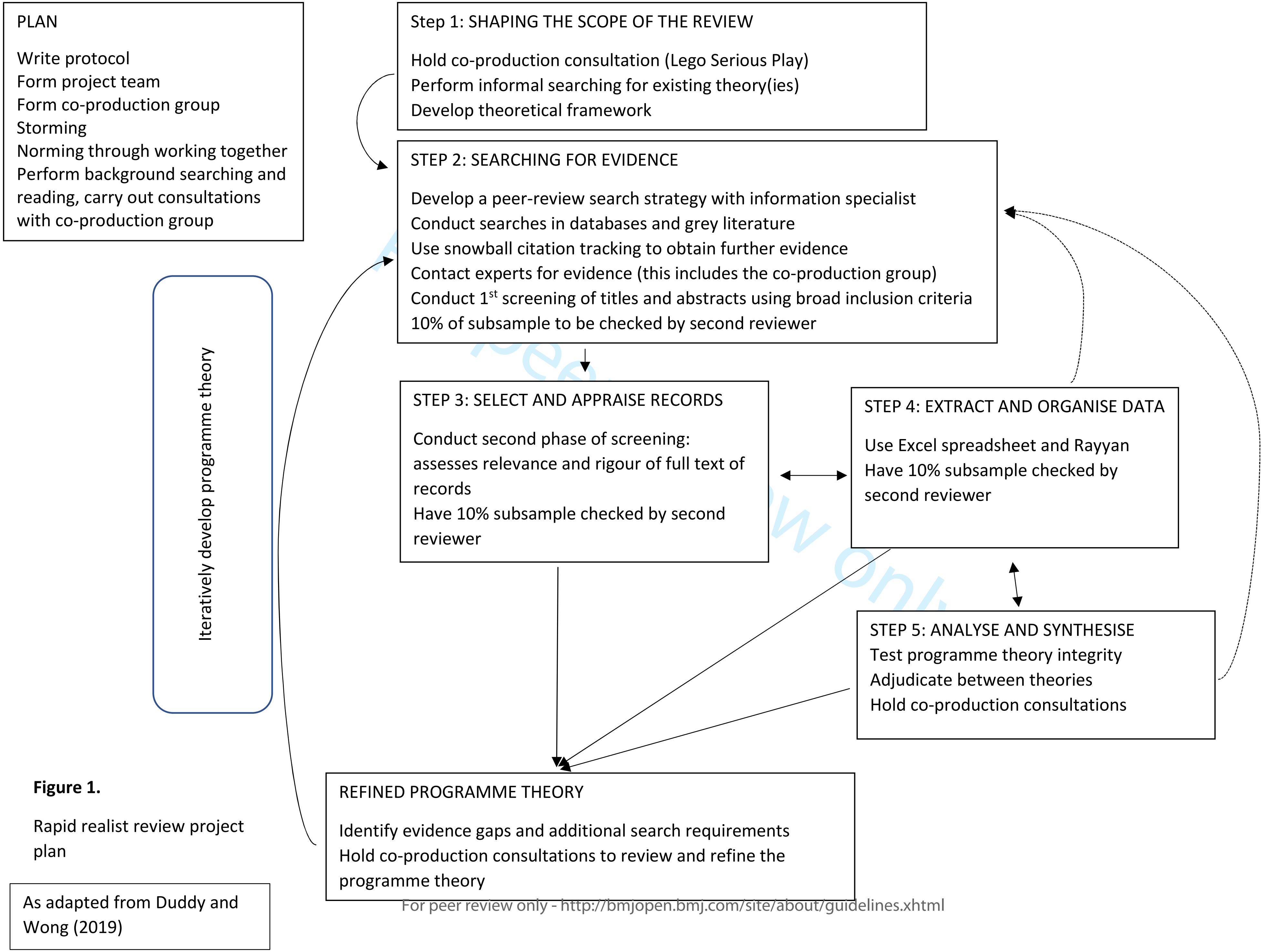

Figure 1.

Rapid realist review project plan

For peer review only - http://bmjopen.bmj.com/site/about/guidelines.xhtml

Supplementary file 1. Positionality statement

The authorship team is based in the UK and consists of individuals from the following backgrounds: academic, clinical, clinical academic, leaders of community organisations, health and social care research delivery, health and social care research funding and leaders of third sector organisations, (government and voluntary). All authors have consented to self-reporting their ethnicity, as indicated on the title page. Some have experience of conducting research, delivering research, funding research, providing healthcare, or support and representation in some way, to people from Black African Diaspora Communities (BAFDC). The co-production group (VE, TK, DG, LH, EM,TO, IS, SW) and one member of the research team (DE) have lived experience of being Black, as defined in the protocol. Two members of the co-production group identify as Black men.The first author (EH) is a White British clinical academic researcher whose work mainly focuses on developing an inclusive health and care research system for individuals from BAFDC. Over the last ten years, she has observed the exclusionary practices, processes and service provision of health and social care research to people from BAFDC through her role as a clinical research nurse in the West Midlands region of England. She has been actively involved as a Patient Public Involvement Lead, contributing to her awareness of the impact of exclusion from health and social care research on the health and well-being of people from BAFDC as well as the difficulties they face in opportunities to take part in health and social care research. She and all co-authors acknowledge the importance of equity, diversity and inclusion, and value an inclusive approach to health and social care research where all individuals, including those from under-served communities, benefit from research opportunities. They will be reflexive throughout this research on their positionality and how this may influence interpretations of people's lived experiences. All authors work as a team and the first author (EH) has regular discussions with the co-production group to ensure the protocol and subsequent review will be led by their cultural and experiential knowledge and expertise.

For peer review only

Figure 2.

Research participation pathway

Pathway of research participation in health and care research for people from Black African Diaspora Communities (BAFDC)

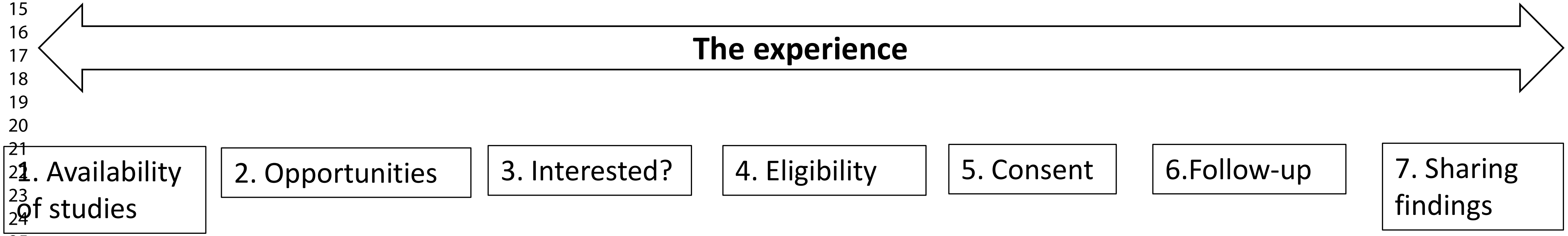

PRISMA 2020 Checklist

Section and Topic	Item #	Checklist item	Location where item is reported
**TITLE**			
Title	1	Identify the report as a systematic review. This is a rapid realist review protocol.	Lines 1-3
**ABSTRACT**			
Abstract	2	See the PRISMA 2020 for Abstracts checklist.	Lines 50-78
**INTRODUCTION**			
Rationale	3	Describe the rationale for the review in the context of existing knowledge.	Lines 257-265
Objectives	4	Provide an explicit statement of the objective(s) or question(s) the review addresses.	Lines 289-298
**METHODS**			
Eligibility criteria	5	Specify the inclusion and exclusion criteria for the review and how studies were grouped for the syntheses.	Table 3 (Line 453 ; Line 456)
Information sources	6	Specify all databases, registers, websites, organisations, reference lists and other sources searched or consulted to identify studies. Specify the date when each source was last searched or consulted.	Table 1 Line 408 + Lines 410 – 415
Search strategy	7	Present the full search strategies for all databases, registers and websites, including any filters and limits used.	Lines 406-421
Selection process	8	Specify the methods used to decide whether a study met the inclusion criteria of the review, including how many reviewers screened each record and each report retrieved, whether they worked independently, and if applicable, details of automation tools used in the process.	Lines 421-434
Data collection process	9	Specify the methods used to collect data from reports, including how many reviewers collected data from each report, whether they worked independently, any processes for obtaining or confirming data from study investigators, and if applicable, details of automation tools used in the process.	Lines 441 - 455
Data items	10a	List and define all outcomes for which data were sought. Specify whether all results that were compatible with each outcome domain in each study were sought (e.g. for all measures, time points, analyses), and if not, the methods used to decide which results to collect.	Lines 458-471
	10b	List and define all other variables for which data were sought (e.g. participant and intervention characteristics, funding sources). Describe any assumptions made about any missing or unclear information.	Lines 458-471
Study risk of bias assessment	11	Specify the methods used to assess risk of bias in the included studies, including details of the tool(s) used, how many reviewers assessed each study and whether they worked independently, and if applicable, details of automation tools used in the process.	Lines 473-475<br>Lines 444-452<br>Lines 422-423
Effect measures	12	Specify for each outcome the effect measure(s) (e.g. risk ratio, mean difference) used in the synthesis or presentation of results.	N/A
Synthesis methods	13a	Describe the processes used to decide which studies were eligible for each synthesis (e.g. tabulating the study intervention characteristics and comparing against the planned groups for each synthesis (item #5)).	Lines 472-482
	13b	Describe any methods required to prepare the data for presentation or synthesis, such as handling of missing summary statistics, or data	Lines 472-

For peer review only

For peer review only - http://bmjopen.bmj.com/site/about/guidelines.xhtml

PRISMA 2020 Checklist

Section and Topic	Item #	Checklist item	Location where item is reported
		conversions.	482
	13c	Describe any methods used to tabulate or visually display results of individual studies and syntheses.	N/A (protocol)
	13d	Describe any methods used to synthesize results and provide a rationale for the choice(s). If meta-analysis was performed, describe the model(s), method(s) to identify the presence and extent of statistical heterogeneity, and software package(s) used.	N/A (protocol)
	13e	Describe any methods used to explore possible causes of heterogeneity among study results (e.g. subgroup analysis, meta-regression).	N/A (protocol)
	13f	Describe any sensitivity analyses conducted to assess robustness of the synthesized results.	N/A (protocol)
Reporting bias assessment	14	Describe any methods used to assess risk of bias due to missing results in a synthesis (arising from reporting biases).	N/A (protocol)
Certainty assessment	15	Describe any methods used to assess certainty (or confidence) in the body of evidence for an outcome.	N/A (protocol)
**RESULTS**			
Study selection	16a	Describe the results of the search and selection process, from the number of records identified in the search to the number of studies included in the review, ideally using a flow diagram.	No results yet as protocol (406-421 describes search strategy
	16b	Cite studies that might appear to meet the inclusion criteria, but which were excluded, and explain why they were excluded.	N/A (protocol)
Study characteristics	17	Cite each included study and present its characteristics.	N/A (protocol)
Risk of bias in studies	18	Present assessments of risk of bias for each included study.	N/A (protocol)
Results of individual studies	19	For all outcomes, present, for each study: (a) summary statistics for each group (where appropriate) and (b) an effect estimate and its precision (e.g. confidence/credible interval), ideally using structured tables or plots.	N/A (protocol)
Results of syntheses	20a	For each synthesis, briefly summarise the characteristics and risk of bias among contributing studies.	N/A (protocol)
	20b	Present results of all statistical syntheses conducted. If meta-analysis was done, present for each the summary estimate and its precision (e.g. confidence/credible interval) and measures of statistical heterogeneity. If comparing groups, describe the direction of the effect.	N/A (protocol)
	20c	Present results of all investigations of possible causes of heterogeneity among study results.	N/A (protocol)
	20d	Present results of all sensitivity analyses conducted to assess the robustness of the synthesized results.	N/A (protocol)
Reporting biases	21	Present assessments of risk of bias due to missing results (arising from reporting biases) for each synthesis assessed.	N/A (protocol)

For peer review only

PRISMA 2020 Checklist

Section and Topic	Item #	Checklist item	Location where item is reported
Certainty of evidence	22	Present assessments of certainty (or confidence) in the body of evidence for each outcome assessed.	N/A (protocol)
**DISCUSSION**			
Discussion	23a	Provide a general interpretation of the results in the context of other evidence.	N/A (protocol)
	23b	Discuss any limitations of the evidence included in the review.	N/A (protocol)
	23c	Discuss any limitations of the review processes used.	Lines 483-505
	23d	Discuss implications of the results for practice, policy, and future research.	Lines 214-215, 244, 279, 504
**OTHER INFORMATION**			
Registration and protocol	24a	Provide registration information for the review, including register name and registration number, or state that the review was not registered.	Not registered
	24b	Indicate where the review protocol can be accessed, or state that a protocol was not prepared.	N/A (protocol)
	24c	Describe and explain any amendments to information provided at registration or in the protocol.	N/A (protocol)
Support	25	Describe sources of financial or non-financial support for the review, and the role of the funders or sponsors in the review.	Lines 552-558
Competing interests	26	Declare any competing interests of review authors.	Line 559
Availability of data, code and other materials	27	Report which of the following are publicly available and where they can be found: template data collection forms; data extracted from included studies; data used for all analyses; analytic code; any other materials used in the review.	N/A (protocol)

From: Page MJ, McKenzie JE, Bossuyt PM, Boutron I, Hoffmann TC, Mulrow CD, et al. The PRISMA 2020 statement: an updated guideline for reporting systematic reviews. BMJ 2021;372:n71. doi: 10.1136/bmj.n71

For more information, visit: http://www.prisma-statement.org/

For peer review only

BMJ Open

Understanding the inclusion and participation of adults from Black African Diaspora Communities (BAFDC) in health and care research in the UK: a realist review protocol.

Journal:	*BMJ Open*
Manuscript ID	bmjopen-2023-082564.R1
Article Type:	Protocol
Date Submitted by the Author:	29-Feb-2024

Complete List of Authors:	Hoverd, Eleanor; University of Warwick Medical School,<br>Effiom, Violet; NIHR Clinical Research Network West Midlands<br>Gravesande, Dionne; Public Contributor<br>Hollowood, Lorna; University of Birmingham<br>Kelly, Tony; PPI, PPI Lead on Project<br>Mukuka, Esther; NIHR<br>Owatemi, Taiwo; UK Parliament<br>Sargeant, Ify; Public Contributor<br>Ward, Shane; Public Contributor<br>Spencer, Rachel; University of Warwick, Warwick Medical School<br>Edge, Dawn; Univ Manchester; The University of Manchester<br>Dale, Jeremy; University of Warwick, Warwick Medical School<br>Staniszewska, Sophie; University of Warwick, Warwick Medical School

**Primary Subject Heading**:	Health services research
Secondary Subject Heading:	Health policy
Keywords:	Clinical Trial, Health Equity, Health Services, Health Services Accessibility, Health policy < HEALTH SERVICES ADMINISTRATION & MANAGEMENT, Patient Participation

SCHOLARONE™
Manuscripts

Title: Understanding the inclusion and participation of adults from Black African Diaspora Communities (BAFDC) in health and care research in the UK: a realist review protocol.

Corresponding Author: Eleanor Hoverd, White British, Unit of Academic Primary Care, Warwick Medical School, Warwick University, Gibbet Hill Rd, Coventry CV4 7AL, England, UK eleanor.hoverd@warwick.ac.uk

Co-authors:

Violet Effiom, Black, Clinical Research Network, West Midlands, UK

Dionne Gravesande, Black British of Caribbean Public Contributor, UK

Lorna Hollowood, Mixed heritage – Black Caribbean and White, School of Nursing and Midwifery, University of Worcester, UK

Tony Kelly, Black British-Caribbean Public Contributor, Birmingham, UK

Dr. Esther Mukuka, Black African (British) Head of Research Inclusion, National Institute for Health and Care Research (NIHR), UK

Taiwo Owatemi, British African, Labour MP, Coventry Northwest, UK

Dr. Ifeanyi Sargeant, Black African Public Contributor, Wrinehill, Staffordshire, UK

Shane Ward, Black Alkebulan/ African Public Contributor, Sandwell, UK

Associate Professor Rachel Spencer, White British, Unit of Academic Primary Care, University of Warwick, UK R.Spencer2@warwick.ac.uk

Professor Dawn Edge, African-Caribbean, Division of Psychology and Mental Health, University of Manchester, UK dawn.edge@manchester.ac.uk

Professor Jeremy Dale, White British, Unit of Academic Primary Care, Warwick Medical School, Warwick University, Coventry, UK jeremy.dale@warwick.ac.uk

For peer review only - http://bmjopen.bmj.com/site/about/guidelines.xhtml

Professor Sophie Staniszewska, White British of Polish origin, DPhil (Oxon), BSc (Hons), Warwick Research in Nursing, Warwick Medical School, Warwick University, Coventry, UK Sophie.Staniszewska@warwick.ac.uk

For peer review only

For peer review only - http://bmjopen.bmj.com/site/about/guidelines.xhtml

Abstract

INTRODUCTION

People from Black African Diaspora Communities (BAFDC) experience poorer health outcomes, have many long-term conditions and are persistently under-represented in health and care research. There is limited focus on programmes, or interventions that support inclusion and participation of people from BAFDC in research. Through co-production, this realist review seeks to provide a programme theory explaining what context and mechanisms may be required, to produce outcomes that facilitate inclusion and participation for people from BAFDC in health and care research, in the UK.

METHODS AND ANALYSIS

A group of people from BAFDC with lived and professional experience, representing all levels of the health and care research system, will co-produce a realist review with a team of African-Caribbean, White British and White British of Polish origin health and care researchers. They will follow Pawson's five steps: 1) shaping the scope of the review; 2) searching for evidence; 3) document selection and appraisal; 4) data extraction; 5) data synthesis. The co-production group will help to map the current landscape, identifying key issues that may inhibit, or facilitate inclusion. Data will be extracted, analysed and synthesised following realist logic analysis, identifying and explaining how context and mechanisms are conceptualised in the literature and the types of contextual factors that exist and impact on inclusion and participation. Findings will be reported in accordance with Realist and Meta-narrative Evidence Synthesis Evolving Standards (RAMESES).

ETHICS AND DISSEMINATION

For peer review only

For peer review only - http://bmjopen.bmj.com/site/about/guidelines.xhtml

The co-production group will agree an ethical approach considering accountability, responsibility, and power dynamics, by establishing a Terms of Reference, taking a reflexive approach and co-producing an ethical framework. Findings will be disseminated to BAFDC and the research community through arts-based methods, peer-reviewed publications and conference presentations, agreeing a co-produced strategy for dissemination. Ethical review is not required.

Prospero registration number CRD42024517124

STRENGTHS AND LIMITATIONS

To our knowledge, this will be the first realist review undertaken exploring the contextual factors that influence inclusion and participation and identification of the mechanisms that facilitate inclusion and participation of people from BAFDC in health and care research, internationally.

Co-producing the review with people from BAFDC with lived and professional experience, will facilitate a deeper understanding of current barriers and facilitators affecting the inclusion and participation of people from BAFDC in health and care research, acting as content experts, drawing on their experiential knowledge which is rarely reported in formal literature.

This study will help to inform the development of an intervention reflected in the methods, that enable greater participation of people from BAFDC in health and care research in the UK and may also be applicable to other health and care research systems.

For peer review only - http://bmjopen.bmj.com/site/about/guidelines.xhtml

A limitation of the review is that the lead researcher (EH) is White British and does not have lived experience of being Black. Thus, she will reflect upon her positionality throughout with support of a diverse research team and Patient Public Mentor who is from a BAFDC.

Keywords Under-representation, Health Inequities, Black People, Participation, Biomedical Research

For peer review only

For peer review only - http://bmjopen.bmj.com/site/about/guidelines.xhtml

INTRODUCTION

Research aims to enhance the health outcomes of the whole population [1, 2]. However, the lack of participation[1] in health and care research studies by under-served groups may contribute to these groups not reaping the benefits of scientific discoveries [3-12]. One of the most affected underserved groups are Black and ethnic minority groups [13].

Studies suggest that Black and ethnic minority populations are most affected by health inequities and have historically been ignored, marginalised, and forgotten in health and care research [13-15]. In the UK, Black African Diaspora Communities (BAFDC) are a minoritised group that includes Black, Black British, Black Welsh, Caribbean or African, and individuals with dual/multiple heritage and other groups who have Black African lineage [16, 17]. Minoritised groups are defined as having their "*cultural, political and social power*" destroyed because of their identity, by structures and processes that uphold power and domination [17].For the purposes of this review, people from BAFDC refers to adults aged ≥18 years, as children and youths from BAFDC, warrants a separate review. Little progress has been made in improving health outcomes for individuals from BAFDC [18-22].

In the UK, Black women are four times more likely to die during pregnancy, or up to one year after giving birth, than White women [23],type 2 diabetes prevalence is up to three times greater in BAFDC [24],one in four Black men will develop prostate cancer [25] , and the prevalence of hypertension and stroke are also higher

[1] Participation in health and care research as defined by the UK's largest funder of research, the National Institute for Health and Care Research (NIHR), is when people take part in a research study [26]. This includes experiences of being recruited into a study, such as a clinical trial, or participating in focus groups, or completing a survey, for example [27].This differs from Patient and Public Involvement Engagement (PPIE) in health and care research, where patients and members of the public are actively involved as partners in the research process across a range of activities in, such as commenting on research materials, or being part of a steering group or contributing to the synthesis of results [26].

For peer review only - http://bmjopen.bmj.com/site/about/guidelines.xhtml

in this population [24, 28, 29]. Such avoidable and unwarranted differences in health, are the culmination of social, economic, environmental, and structural disparities caused by the unequal distribution of power and resources, underpinned by structural inequalities and racism [14, 30]. However, it remains unclear as to how genetic determinants may contribute to the higher incidence of certain diseases amongst people from BAFDC, primarily due to a lack of representation of these populations in genomics research which may have potentially serious consequences on treatment decisions [31,32]. For example, doses for the drug warfarin, a blood-thinning drug, have been reported as being prescribed based upon an individual's race and ethnicity, despite genetic studies mainly being conducted on European and White Americans [31]. There is also evidence to suggest that there is some misunderstanding amongst biomedical researchers around the differences between sociopolitical constructs like race and ethnicity and biological factors such as genetic ancestry, which can lead to estimates of genetic heredity being biased if environmental and social determinants of health are taken into consideration during analysis [33]. Enabling representation of people from BAFDC in health and care research is critical for innovation and new discoveries; data science; ensuring access to specific therapeutic drugs, or treatments; building trust in science and medicine; addressing health disparities and ensuring provision of evidence-based healthcare [12, 34]. Without this, there is a risk of perpetuating health inequalities and social injustice with data producing algorithms used to guide treatment decisions based on White populations [34]. Furthermore, the potential benefits of personalised medicine and access to new treatments such as in cancer trials may not be realised if people from BAFDC are excluded from research [35].

For peer review only - http://bmjopen.bmj.com/site/about/guidelines.xhtml

The responsibility to improve participation has often been directed at individuals who are under-represented. Terms such as “hard-to-reach”, have been frequently used to describe individuals who are marginalised, or disadvantaged, creating an unhelpful narrative that places the blame on individuals, as opposed to services being inaccessible due to a number of barriers such as studies not routinely reflecting the needs of under-served groups [36, 37]. Health and care research is crucial for reducing health disparities and improving health and well-being globally, but without diverse participation and the representation of all groups, especially those who have some of the poorest health outcomes, social equity cannot be reached, creating an enduring challenge for some of the wealthiest, research-active countries [2, 12, 21].

The science of inclusion

In comparison to the UK, the United States has had a lengthy history of policies implemented to improve diversity within health and care research; consequently, the evidence base around inclusion and participation of Black and ethnic minority populations is largely from the US [12, 22, 38-45]. A report by The National Academies of Sciences Engineering and Medicine (NASEM) (2022) suggests that these policies have failed to reduce exclusion of under-served populations [12]. After the implementation of the *National Institute of Health (NIH) Revitalisation Act of 1993*, there was some reported increase in previously excluded groups, such as women, taking part in health and care research, though individuals from Black and ethnic minority populations remained under-represented [44-47]. Guidance around improving inclusion is problematic when attempting to operationalise, reproduce or measure it [48, 49]. Adoption of NIH policies have not adequately encouraged researchers to widen their eligibility criteria, with the Federal Drugs Agency lacking the power to enforce recommendations, resulting in exclusion of the very groups the

For peer review only - http://bmjopen.bmj.com/site/about/guidelines.xhtml

Act was developed [12]. Exclusion has been related to the lack of reporting of outcomes in research studies by race and ethnicity, impacting upon the evidence-base for clinical decision-making [12]. Exclusion is reported to have been observed in commercial cancer trials, where eligibility criteria can be very restrictive [50-52]. These trials often exclude individuals with comorbidities, which are more prevalent in Black and ethnic minority populations, preventing access to new treatments, such as immunotherapy [51, 52]. The impact of eligibility criteria on inclusion requires further investigation to find solutions. Consequently, a move to develop a stronger body of knowledge about the science of inclusion is emerging, focussing on theory and empirical evidence related to the process of health and care research from initial engagement with individuals from Black and ethnic minority populations to participation in studies [12].

In the UK, the National Institute for Health and Care Research (NIHR) defines research inclusion as "*taking a whole systems approach to what we do and how we do it; identifying and removing longstanding, structural barriers to success across our people, policies, processes and practices*" [53].Robust evidence is needed to inform how the deficits in the infrastructure of health and care research systems should be addressed in order to focus on inclusion and participation [54].There is a need to understand the contextual and causal factors that influence inclusion and participation and to develop the science of inclusion based on theory; clear explanations about theories of inclusion and participation of Black and ethnic minority populations are lacking [12, 54].

The importance of inclusion

In the UK, there has been less focus on policy measures around inclusion in health and care research (for example, there is a lack of practice and policy in regards to

For peer review only - http://bmjopen.bmj.com/site/about/guidelines.xhtml

reporting of inclusion and assessments of quality of data collection), and more focus on recognising the importance of inclusion, and working to identify and address barriers to inclusion [24,53]. This contrast may reflect fundamental differences in the values and politics of the UK and US and the long history of race and ethnicity data collection policies and standards in the US [48,55,56]. The UK's National Institute for Health and Care Research (NIHR) has acknowledged the importance of inclusion in health and care research by developing and implementing a *Research Inclusion Strategy 2022-2027* demonstrating commitment to a long-term inclusivity agenda [53] . A focus on widening access and participation for greater diversity and inclusion is a critical aspect of the strategy, indicating that "*work is required to initiate more inclusive recruitment strategies that promote participation from under-served groups nationwide*" [53]. Creating a more equitable health and care research system is crucial to ensuring disadvantaged groups most affected by health inequities benefit from participating in research and feel that research is relevant to them [24, 39].

Lack of progress

Globally, little progress has been made on participation of Black populations in research studies [12]. There is strong evidence in the US to suggest that African Americans are willing to participate in research, and the decision to participate may be influenced by mechanisms situated at other levels of the health and care research system (such as the intrapersonal and structural levels, rather than at an individual level) [12, 49]. There are reports that willingness to participate has been misrepresented due to biases held by researchers, though the mechanisms that cause bias to function at the intrapersonal and structural levels of health and care systems are poorly understood [12, 49]. It has been suggested that the responsibility for developing interventions that will improve inclusion and participation of people

For peer review only - http://bmjopen.bmj.com/site/about/guidelines.xhtml

from BAFDC lies with researchers, institutions, decision-makers, policymakers and funders of research [12, 57]. Such interventions are required at multiple levels of the health and care research system including policymaking and funding to provide resources to a specific population with the aim of creating progressive change [40]. The way in which an intervention is developed and implemented is dependent upon the power of individuals and institutions and how they manage its implementation, which is pertinent when considering the architecture required for an intervention, for a marginalised population such as BAFDC [58]. For example, if an intervention is designed and implemented by individuals from a perceived dominant population, it may lack the perspectives of a diverse population which may impact upon the success of its design [13]. For an intervention to have the best chance of success, it is crucial to understand the context within which it will be implemented, as well as the mechanisms by which it works. Consequently, a realist approach provides the most suitable method for developing new thinking through offering a deeper, more pragmatic explanation than a systematic review [59]. While systematic reviews offer an assessment of the effectiveness of interventions, they often lack explanations as to how and why an intervention may, or may not have worked, which is particularly valuable in complex systems, such as the health and care research system[59] . The learning from this realist review will be a timely addition to existing efforts. We are at a pivotal point in addressing the structural, racial injustices that have been brought to the fore by critical events over recent years, such as the murder of George Floyd and the COVID-19 pandemic that disproportionately impacted Black and ethnic minority communities [14, 60,61].

METHODS AND ANALYSIS

Our positionality statement can be found in online supplemental file 1.

For peer review only - http://bmjopen.bmj.com/site/about/guidelines.xhtml

Aims

This realist review will broadly explore secondary data to provide insights that will contribute to the development of a realist theory of inclusion and participation in health and care research by people from BAFDC in the UK. The theory may be applicable to other health and care research systems internationally. It will be undertaken with a co-production group composed of people from BAFDC who have the lived experience of being Black as well as experience as: patients, members of the public, healthcare professionals, community leaders, health and care researchers, research delivery staff, policymakers and funders of health and care research. Thus, they are aware of the challenges related to inclusion and participation in health and care research for people from BAFDC [62]. A realist review benefits from “content experts” such as the co-production group, improving the efficiency of the review through helping to focus the scope of the review, development of the initial programme theory and refining the search process and accurate interpretation of the results [63]. The review will help to explain the context of the health and care research system, identifying underlying causal factors that influence inclusion and participation of people from BAFDC in health and care research. We will develop a programme theory through following five key steps as recommended by Pawson et al (2005) that explains how inclusion and participation may be facilitated with people from BAFDC in health and care research [64,65]. This programme theory is intended to inform the development of an intervention to improve inclusion and ultimately outcomes for people from BAFDC.

Review questions

1. What are the contextual and/or causal factors that influence inclusion and participation in health and care research by people from BAFDC?

For peer review only - http://bmjopen.bmj.com/site/about/guidelines.xhtml

2. How and where do mechanisms occur that underly implicit and complicit bias that affects the inclusion and participation of people from BAFDC in health and care research?
3. What barriers and facilitators do people from BAFDC feel affects their experiences of participation in health and care research?
4. What might the components of an intervention(s) look like that fosters inclusion and participation of people from BAFDC in health and care research?

Study design

The design will be guided by Pawson et al.'s five steps and in accordance with RAMESES for quality and reporting [66]. Figure 1 shows a diagram of the review process as adapted from Duddy and Wong [67]. Due to the complexity of context, the focus of the review will be steered by a unique theoretical framework developed with the co-production group from pre-existing mid-range theories deemed pertinent to this research. Theories will be identified through analysis of resources from the review team, as well as comprehensive evaluation of the literature [59]. A realist review is a theory-driven method for investigating complex interventions, or programmes [65]. Evidence is produced in the form of theories that are best understood by analysing the contexts, mechanisms and outcomes of a programme [59]. Understanding context can help to create an optimal condition for the successful implementation of a programme or intervention [68]. The interaction between context (C) and mechanism (M) results in an outcome (O) [69]. This formula is known as a CMO configuration (CMOC) and is a way of explaining theory in realist research [59,69].

INSERT Figure 1

For peer review only - http://bmjopen.bmj.com/site/about/guidelines.xhtml

Patient and Public Involvement

Stakeholder involvement is key to realist research and will be achieved by using a co-production approach to patient and public involvement [69]. Stakeholders are people who may have an interest in the research being carried out, may be involved in shaping it and could be affected by the outcomes [70]. Co-production is a way of sharing power, taking into account the skills and many perspectives within a group of people - it involves respecting and valuing the knowledge of others as well as providing reciprocity and focussing on building and maintaining relationships [71].

Recruitment

A co-production group of eight individuals (VE, TK, DG, LH, EM, TO, IS, SW) from BAFDC will meet to share knowledge and identify training needs, around the realist approach, prior to the review. Individuals were approached by the lead author (EH) through her existing networks (some had already been involved in Patient Public Involvement during the development phase of the research proposal): directly approaching Black community organisations and individuals, providing written information about co-production and the research project, with 1:1 meetings held virtually with all who were interested, to provide an opportunity to ask questions and meet EH. The co-production group includes Experts by Experience, as well as stakeholders from the health and care research system who self-identify as belonging to BAFDC. In this review, Experts by Experience are regarded as patients and members of the public with lived experience of the health and care research system [72].

Work schedule

We will map out the review process and draw on expertise from within the group, through creative methods and discussion, to understand how the health and care

For peer review only - http://bmjopen.bmj.com/site/about/guidelines.xhtml

research system currently serves people from BAFDC in the UK. The co-production group will be consulted in alignment with the principles of co-production throughout, as key “experts” with additional stakeholders sought for expertise, if required [70]. The group will be consulted throughout the review via virtual meetings on Microsoft Teams and email, allowing for wider geographic inclusion as the group is based in the West Midlands and London regions, improving accessibility, with sessions kept to a maximum of 1½ hours to avoid digital fatigue [73].There will also be at least one face-to-face meeting, held at a central location convenient to the group, to allow for longer discussion and to help build relationships.

Sequence of steps

The review will be conducted following five steps: 1) shaping the scope of the review 2) searching for the evidence 3) document selection and appraisal, which will be based on relevance and rigor 4) data extraction and organisation of evidence will be conducted iteratively 5) data synthesis will be used to explain deficits in context and how they impact on inclusion and participation and development of programme theory.

Step 1: Shaping the scope of the review: key concepts and construction of a theoretical framework

Programme theories create a scaffolding to explore how, why, for whom and under what circumstances complex programmes, or interventions work [74].In realist terms, an intervention is a theory [74].This stage will focus on the development of a theoretical framework that will shape the focus of the review, support data extraction, appraisal and synthesis, and identify concepts around inclusion and participation of people from BAFDC in health and care research [71]. Evidence suggests that a small number of theories can be developed into a framework that will support the

For peer review only - http://bmjopen.bmj.com/site/about/guidelines.xhtml

development of initial programme theory that incorporates the multiple levels of a system [75]. To identify relevant theories, concepts critical to inclusion and participation of people from BAFDC will be identified through creative discussion with the co-production group using Lego Serious Play (LSP) as a method for facilitating their input [76]. A small pilot with NIHR Research Champions, or members of the public who have experience of being involved and engaged in health and care research will take place prior to using the approach with the co-production group.

Outputs

We will produce a simple, research participant pathway representing the health and care research process that will be used as an artifact, to consult with the co-production group to stimulate discussion during LSP, to support the facilitation of individual's thoughts and reasoning [77] (see Figure 2).

INSERT Figure 2

The co-production group will use LSP to map the existing health and care research system. Concepts from other programmes in related areas can also be drawn upon. Existing mid-range theories will then be identified through an initial scoping search based upon key concepts that arise following the LSP activity. A list of these mid-range theories will be presented to the co-production group to decide whether they should be included in the theoretical framework, based upon their importance to the research aim and relevance to people from BAFDC. Theories deemed pertinent by the group will be merged into a framework to guide data extraction and appraisal. Evidence will be collected to theorise how an intervention may work, facilitating identification of CMOC's through matching to existing theories, while considering information that may be missing from existing mid-range theories [59, 78].

Step 2: Searching for evidence

For peer review only - http://bmjopen.bmj.com/site/about/guidelines.xhtml

A search for evidence will be conducted, including international literature, collecting a wide range of secondary data sources and grey literature including: all types of studies, peer-reviewed articles, opinion pieces, commentaries, conference papers/proceedings, reports, and social media excerpts [62,65]. In discussion with an Information Specialist a pilot search of the evidence was conducted. The review will be conducted over a six-month period including all literature that covers research that addresses the issues of inclusion and participation in health and care research in the period post World War II. There are no language restrictions. We expect further adjustments to the search strategy to be required. Discussions and decision-making with the co-production group will be held around relevance of international literature to a UK context. A data extraction form, will be informed by the theoretical framework as a way of combing the evidence and appraising it through scoring it as high, moderate, low based upon its usefulness and relevance [79]. The data extraction form will include standard information including: full reference, item type, country where source was published, quality and the group being researched, though will be developed iteratively to meet the needs of the review [79].

Search terms:
Health Services Research
Clinical Trials
Biomedical Research
Black
Afro-Caribbean

Data sources and search strategy

An initial search will be developed based on the theoretical framework, with key search terms identified in Table 1. An example of the search strategy for Medline can be found in supplemental file 2. *EndNote* will be used for storing all identified sources [80]. Search terms will be discussed with the research team and co-production group and modified as required for each database, or website.

For peer review only

BME
BAME
[illegible]
Participation, or Patient Participation, or Community Participation, or Stakeholder Participation, or Social Participation
Inclusion
Recruitment
Underrepresentation

**Initial grey literature sources**
NHS Race and Health Observatory
Runnymede Trust
BRAP
The Health Foundation
The King's Fund
Operation Black Vote
The Voice newspaper
African Voice newspaper
West Bromwich African Caribbean Resource Centre
Enfield Caribbean Association
CAHN (Caribbean and African Health Network)
Vanderbilt University Medical Centre, Office of Health Equity Anti-racism hub
CARE (Community, Access, Recruitment and Engagement) Center, Massachusetts General Hospital
Open Access Theses and Dissertations

Table 1. Search terms

Using the strategy, a search for the evidence will be conducted using the following databases: Medline, EMBASE, PsycINFO, Web of Science, Race Relations Abstracts, Sociological Abstracts, University of the West Indies, and Patient-Centred Outcomes Research Institute (PCORI). A search of grey literature will be conducted (Table 2), although recommendations from the co-production group may also evolve as the review progresses. Literature sources will be prioritised by the co-production group where relevant.

For peer review only

Caribbean-studies@jiscmail.ac.uk
Caribbeanintelligence
Twitter, Facebook and YouTube
Co-production group recommendations

Table 2. Grey literature sources

Once the initial search has been completed, we will decide if further iterative searching will be conducted which will be determined by the extent of literature identified and discussed with the research team and co-production group. Search terms will be expanded in this instance with the information specialist.

Screening of abstracts will be carried out according to inclusion and exclusion criteria developed, based upon the theoretical framework, with no date limit initially. This will be reviewed following the database search and a limit set, should the return of documents be excessive.

Screening process

EH will undertake screening based on title and abstract and a second reviewer will screen a random sample of 10% of the identified citations, setting a kappa measure of k> 0.8 to measure inter-rater reliability [66]. This will be calculated using the formula for Cohen's kappa [81]. Reviewers will discuss disagreements with the co-production group and research team. If k<0.8 a further 10% sample will be screened, and the outcome discussed with the research team. Should an abstract not be available (for example, in some grey literature), documents will be included and full screening of each will be carried out at second level screening. *Rayyan* referencing software will be utilised for screening as it enables blinding of screening decisions, collaboration functions and presentation of screening decisions in a simple format which can be exported onto a spreadsheet [82]. Papers that are excluded by one

For peer review only - http://bmjopen.bmj.com/site/about/guidelines.xhtml

reviewer and included by another will be brought to the wider research team for further discussion, as well as those where one, or both reviewers are unsure.

Iterative searching

As realist reviews are iterative, searching for additional data to support theory development is likely [59]. For each additional search, the research team will develop further inclusion/exclusion criteria, with each search tested and refined through support from an Information Specialist and screening conducted as summarised above.

Step 3: selection and appraisal

After initial screening in step 2, documents will be selected based upon relevance and rigour after the full text has been read by EH and inclusion/exclusion criteria applied [83] (see Table 3). Relevant documents are those which are deemed helpful and valuable for building theory whilst rigour ascertains the trustworthiness and credibility of methods used to produce data [84]. Although, methodological quality of papers is not so important in realist research, relevance and robustness is [85]. Evidence may include a wide range of sources e.g. opinion pieces that may capture important evidence based upon experiences [83].Pawson asserts that even poor evidence, which may be methodologically weak, can provide rich insights and thus following a hierarchical approach to selecting documents should not pertain to realist reviews [64, 83, 84]. Realist research is informed by evidence, as opposed to evidence-based and therefore a diverse range of literature sources are needed to develop programme theories with sources selected according to relevance and rigour [85].

Table 3. Inclusion and exclusion criteria

**Inclusion**

For peer review only - http://bmjopen.bmj.com/site/about/guidelines.xhtml

No initial date parameter.<br><br>All study designs (qualitative, mixed methods, quantitative, systematic reviews etc…) and grey literature.<br><br>All international sources will be included.<br><br>Document has relevance to development of Programme Theory, either the full text, or a section.<br><br>Population must be related to Black African Diaspora Communities (BAFDC), Black British, African American, African or African-Caribbean people, though this may include sources that include information about other ethnic minority groups as well.<br><br>Must concern factors that affect inclusion and participation in health and care research with health and social care research.
**Exclusion**
Relates to BAFDC, Black British, African American, African, African-Caribbean people <18 years old.

Step 4: extracting and organising data

Databases will be developed to extract and organise data, tailored for this review. Data will be extracted by EH with a 10% subsample of included papers, also completed by the second reviewer. Disagreements will be discussed with the research team and co-production group. If kappa is <0.8 then a further sub-sample of 10% will be reviewed by the second reviewer. We will pilot the use of *NVivo* software as an alternative to *Excel* to assist data coding and organisation; we will code three to five documents in both databases to determine which is the most pragmatic tool for this review [86]. Extracted data will be centred on dialogue that helps to build programme theory [87]. Codes will be developed both inductively from documents, and deductively from the theoretical framework [88]. Data will be organised into broad, conceptual categories , through grouping the codes together and prioritising coding for causal insight in regards to the architecture of the health and care research system [89]. This will support identifying patterns in the data and configuring data into CMOCs [88] .

For peer review only

Step 5: analysis and synthesis

Data analysis and synthesis will be led by EH with reflections and discussion amongst the research team and co-production group to support interpretation of CMOC's using retroduction [90]. Retroduction allows the researcher to incorporate intuitions and insights, as well as inductive and deductive logic, considering the causal forces that may be producing what is observed [90]. This theorising is based upon the researchers' interpretations and may not be testable in the first instance, particularly if only part of a theory is developed [90]. The co-production group will be involved in co-producing this, to ensure that the developing programme theory, is true to their lived and professional experiences. The group will decide meeting frequency, location, and length.

Strengths and limitations

A key strength of this realist review lies with the co-production group that will be involved throughout [62]. The combined skills and expertise of a group of stakeholders from BAFDC, with both lived and professional experience in the health and care research system will place people from BAFDC at the centre of this review. The group's lived experience of being Black, or having a shared history of "*colonialism and enslavement in the past and continuing to experience racism and diminished opportunities*" makes them experts in their own right and will ensure that their reality cannot be denied [91]. A limitation is that the lead researcher (EH) is White British and has not experienced racism. Therefore, she will reflect on her positionality throughout, with a willingness to engage, learn, and take on board the complex issues that may present. The co-production group feel that Tuckman's model of "*forming, storming, norming and performing*" is a useful pathway for reflecting on how the group may develop to ensure the aims and objectives of this

For peer review only - http://bmjopen.bmj.com/site/about/guidelines.xhtml

project are met and to create a progressive group culture [92]. This review will be undertaken as a realist review, in order to answer timely research questions for which there is limited published evidence on interventions or programmes that effectively foster inclusion and participation in health and care research with people from BAFDC [58, 63]. Limitations may include not being able to access or include all sources identified in searches as well as a limit on the number of iterative searches conducted due to timescales of the research. However, together as a research team and co-production group, we will agree on the priorities of the programme theory with a view to conducting future research on additional aspects that we are unable to address in this project.

ETHICS AND DISSEMINATION

This review does not require ethical approval due to the use of secondary data that already exists e.g. in databases and Patient Public Involvement (PPI) in the form of co-production. The review is registered with PROSPERO. However, ethical considerations around accountability, responsibility and power dynamics will be discussed and reflected upon, with the co-production group and research team. This review will lead to a programme theory that begins to explain what may work, for supporting inclusion and participation of people from BAFDC in health and care research internationally. This theory will continue to be built through qualitative data collection methods, with stakeholders from each level of the health and care research system, in a realist evaluation. Further outputs will include a set of principles, recommendations and a conceptual framework that will inform the development of an intervention suitable for implementation, to support a UK research system focussed on equity and social justice [12]. An early prototype for an intervention, based on the findings, will be developed with the co-production group.

For peer review only

For peer review only - http://bmjopen.bmj.com/site/about/guidelines.xhtml

Outputs for people from BAFDC will be co-produced and will focus on a series of community engagement events, dissemination of findings via radio, in newspapers such as *The Voice*, a well-established British African Caribbean newspaper since 1982 and via social media.

Figure 1. Realist review project plan

Figure 2. Pathway of experience of participation

Table 1. Search terms

Table 2. Grey literature sources

Table 3. Inclusion Exclusion criteria

Authors' contributions EH conceived the study with input from SS, DE, JD, RS and TK. EH was responsible for the design and drafting of the protocol. SS, DE, JD, RS, TK, SW, EM, IS, VE, TO, LH, DG contributed to protocol development and SS, DE, JD, RS also provided methodological advice. SS, DE, JD, RS, TK, SW, EM, IS, VE, TO, LH, DG provided criticism and refinement of the manuscript. SS, DE, JD, RS, TK, SW, EM, IS, VE, TO, LH, DG approved the final version.

Funding statement EH is funded by the NIHR [Health Education England/National Institute for Health and Care Research Clinical Doctoral Research Fellowships programme, grant number 302121]. SS is part funded by the NIHR Applied Research Collaboration (ARC) West Midlands [grant number NIHR200165], the NIHR Health Protection Research Unit (HPRU) Gastrointestinal Infections [grant number NIHR200910], the NIHR HPRU Genomics and Enabling data [grant number NIHR200892], the NIHR West Midlands Evidence Synthesis Group [grant number NIHR153453] and the NIHR Health Determinants Research Collaboration Coventry [Not yet awarded]

Competing interests There are no competing interests.

For peer review only

References

1. Gov.uk. Saving and Improving Lives: The Future of UK Clinical Research Delivery [online].2021. https://www.gov.uk/government/publications/the-future-of-uk-clinical-research-delivery/saving-and-improving-lives-the-future-of-uk-clinical-research-delivery (accessed 18 December 2022)

2. WHO. WHO's goal : Forward looking and prioritised global health research [online].2023. https://www.who.int/our-work/science-division/research-for-health (accessed 3 April 2023).

3. Iyizoba-Ebozue Z, Fatimilehin A, Mbanu P et al Reflection on Black and Ethnic Minority Participation in Clinical Trials. Clinical Oncology (Royal College of Radiologists), 2022; 34(10):674-677. doi:10.1016/j.clon.2022.07.006

4. Smart A and Harrison E The under-representation of minority ethnic groups in UK medical research. Ethn Health, 2019;22(1): 65-82. doi: 10.1080/13557858.2016.1182126

5. Treweek S, Forouhi N, Narayan K et al COVID-19 and ethnicity: who will research results apply to? The Lancet, 2020; 95(10242):1955-1957. doi:10.1016/S0140-6736(20)31380-5

6. Sheppard V, Sutton A, Holmes E et al Recruitment of African Americans into Cancer Clinical Research: Strategies and Outcomes. Journal of Urban Health, 2021;98(Suppl 2):149-154. doi:10.1007/s11524-021-00565-8

7. Lau Y, Podlewska A, Ocloo J et al Does Ethnicity Influence Recruitment into Clinical Trials of Parkinson's Disease? Journal of Parkinsons Disease, 2022;12(3):975-981. doi:10.3233/JPD-213113

8. Iqbal H, West J, Haith-Cooper M et al A systematic review to identify research priority setting in Black and minority ethnic health and evaluate their processes. PLoS ONE [Electronic Resource], 2021. 16(5): p. e0251685. doi.org/10.1371/journal.pone.0251685

9. Cunningham-Erves J, Mayo-Gamble T, Hull P et al A pilot study of a culturally-appropriate, educational intervention to increase participation in cancer clinical trials among African Americans and Latinos. Cancer Causes & Control, 2021; 32(9):953-963. doi:10.1007/s10552-021-01449-7

10. De las Nueces D, Hacker K, DiGirolamo A et al A systematic review of community-based participatory research to enhance clinical trials in racial and ethnic minority groups. Health Services Research 2012 47(3 Pt 2):1363-1386. doi:10.1111/j.1475-6773.2012.01386.x

11. McFarlane S, Peng S, Awonuga O et al Community-Based Participatory Research (CBPR) to Enhance Participation of Racial/Ethnic Minorities in Clinical Trials: A 10-Year Systematic Review, Health Communication.2022; 37(9):1075-1092. doi:10.1080/10410236.2021.1943978

For peer review only - http://bmjopen.bmj.com/site/about/guidelines.xhtml

12. National Academies of Sciences, Engineering, and Medicine (NASEM) Improving Representation in Clinical Trials and Research : Building Research Equity for Women and Underreprsented Groups, ed. K.B.-D.a.A. Helman. Washington DC: National Academies Press 2022

13. Williams D, Lawrence J, Davis B et al Racism and Health: Evidence and Needed Research. Annu Rev Public Health, 2019; 40:105-125. doi:10.1146/annurev-publhealth-040218-043750

14. Nobles M, Womack C, Wonkham A et al RACISM Overcoming science's toxic legacy.. 2022 Nature Overcoming racism in science: A Nature special issue 2022 Jun;606(7913):225-227. doi: 10.1038/d41586-022-01527-z.

15. Lewis T, Buck D, Wenzel L Equity and endurance: how can we tackle health inequalities this time? The King's Fund (kingsfund.org.uk) [online] 2022. https://www.kingsfund.org.uk/insight-and-analysis/long-reads/how-can-we-tackle-health-inequalities (accessed 28 Mar 2023)

16. Knight, H, Deeny, S, Dreyer, K et al Challenging racism in the use of health data. The Lancet Digital Health, 2021; 3(3):e144-e146. doi:10.1016/S2589-7500(21)00019-4

17. Selvarajah S, Deivanayagam G, Lasco S et al Categorisation and Minoritisation. BMJ Global Health, 2020; 5(12):e004508. doi:10.1136/bmjgh-2020-004508

18. Birmingham City Council. Birmingham and Lewisham African Caribbean Health Inequalities Review (BLACHIR).[online] 2022; https://www.birmingham.gov.uk/downloads/file/23111/blachir_report (accessed 5 Feb 2024).

19. Petersen, J., J. Kandt, and P.A. Longley, Ethnic inequalities in hospital admissions in England: an observational study. BMC Public Health, 2021. 21(1): p. 862. doi.org/10.1186/s12889-021-10923-5

20. Mukadam, N, Marston L, Lewis G, et al., Incidence, age at diagnosis and survival with dementia across ethnic groups in England: A longitudinal study using electronic health records. Alzheimers Dement, 2023. 19(4): p. 1300-1307. doi: 10.1002/alz.12774

21. Kapadia D, , Salway S, Nazroo J, et al Ethnic Inequalities in Healthcare: A Rapid Evidence Review.[online] 2022; https://www.nhsrho.org/wp-content/uploads/2023/05/RHO-Rapid-Review-Final-Report_.pdf.(accessed 5 Feb 2024)

22. Awidi M and S Hadidi Participation of Black Americans in Cancer Clinical Trials: Current Challenges and Proposed Solutions. JCO Oncology Practice, 2021;17(5):265-271. doi:10.1200/OP.21.00001

23. MBRRACE-UK. MBRRACE-UK Perinatal Mortality Surveillance: UK Perinatal Deaths for Births from January to December [online] 2021

For peer review only - http://bmjopen.bmj.com/site/about/guidelines.xhtml

https://www.npeu.ox.ac.uk/mbrrace-uk/reports/perinatal-mortality-surveillance (accessed 2 Feb 2023)

24. Raleigh V The King's Fund.The health of people from ethnic minority groups in England [online] 2023. The Health Of People From Ethnic Minority Groups In England https://www.kingsfund.org.uk/insight-and-analysis/long-reads/health-people-ethnic-minority-groups-england (accessed 5 Feb 2024)

25. Prostate Cancer UK. Prostate cancer and other prostate problems: Information for black men [online] 2023 https://shop.prostatecanceruk.org//our-publications/all-publications/prostate-cancer-and-other-prostate-problems-information-for-black-men?limit=100 (accessed 8 Jul 2023)

26. NIHR. Glossary [online] 2023 https://www.nihr.ac.uk/about-us/glossary.htm?letter=P&postcategory=-1 (accessed 11 May 2023)

27. NIHR. Briefing notes for researchers - public involvement in NHS, health and social care research [online] 2021 https://www.nihr.ac.uk/documents/briefing-notes-for-researchers-public-involvement-in-nhs-health-and-social-care-research/27371 (accessed 28 Jan 2023)

28. Su D, Yang H, Chen Z et al Ethnicity-specific blood pressure thresholds based on cardiovascular and renal complications: a prospective study in the UK Biobank. BMC Medicine, 2024. 22(1): p. 54. doi:10.1186/s12916-024-03259-5

29. Ali R, Chowdhury A, Forouhi N et al Ethnic disparities in the major causes of mortality and their risk factors - a rapid review. [online] 2021,.Commission on race and ethnic disparities. https://www.gov.uk/government/publications/the-report-of-the-commission-on-race-and-ethnic-disparities-supporting-research/ethnic-disparities-in-the-major-causes-of-mortality-and-their-risk-factors-by-dr-raghib-ali-et-al (accessed 5 Feb 2024)

30. Rai T, Hinton R, McManus et al What would it take to meaningfully attend to ethnicity and race in health research? Learning from a trial intervention development study. Sociology of Health & Illness, 2022; 44 Suppl 1(Suppl 1):57-72. doi:10.1111/1467-9566.13431

31. Cerdeña, J., Grubbs V, . Non A, Racialising genetic risk: assumptions, realities, and recommendations. The Lancet, 2022. 400(10368): p. 2147-2154.doi: 10.1016/S0140-6736(22)02040-2.

32. Fatumo, S, Chikowore T, Choudhury A et al,, A roadmap to increase diversity in genomic studies. Nature Medicine, 2022. 28(2): p. 243-250.doi: 10.1038/s41591-021-01672-4

33. Phuong, J, Riches, N, Madlock-Brown, C, et al (2022), Social Determinants of Health Factors for Gene–Environment COVID-19 Research: Challenges and Opportunities. *Advanced Genetics* 3: 2100056. doi.org/10.1002/ggn2.20210005633

For peer review only - http://bmjopen.bmj.com/site/about/guidelines.xhtml

34. Knight H, Deeny S, Dreyer K et al Challenging racism in the use of health data. The Lancet Digital Health, 2021; 3(3):e144-e146. doi:10.1016/S2589-7500(21)00019-4

35. The Lancet. Personalised medicine in the UK.2017; 391(10115): e1 doi.org/10.1016/S0140-6736(17)33261-0

36. NSPCC. Why language matters: reframing responsibility for accessing services [online] 2022 https://learning.nspcc.org.uk/news/why-language-matters/not-hard-to-reach-reframing-responsibility-for-accessing-services (accessed 15 Apr 2023).

37. Flanagan S and Hancock B 'Reaching the hard to reach' - lessons learned from the VCS (voluntary and community Sector). A qualitative study. BMC Health Services Research, 2010; 10:92. Published 2010 Apr 8. doi:10.1186/1472-6963-10-92

38. Rosen C, Cook N, Wilkins C et al Diversity in Clinical Trials — Next Steps. New England Journal of Medicine, 2022. 387(15) e34 doi:10.1056/nejmp2201379

39. Dawson S, Campbell S, Giles Set al Black and minority ethnic group involvement in health and social care research: A systematic review. Health Expectations, 2018; 21(1):3-22. doi:10.1111/hex.12597

40. George S, Duran N, Norris K et al A systematic review of barriers and facilitators to minority research participation among African Americans, Latinos, Asian Americans, and Pacific Islanders. Am J Public Health, 2014;104(2):e16-e31. doi:10.2105/AJPH.2013.301706

41. Camidge D, Park H, Smoyer K et al Race and ethnicity representation in clinical trials: findings from a literature review of Phase I oncology trials. Future Oncology, 2021; 3271-3280. doi:10.2217/fon-2020-1262

42. Kwiatkowski K, Coe K, Bailar J et al Inclusion of minorities and women in cancer clinical trials, a decade later: Have we improved? Cancer, 2013; 119(16):2956-2963. doi:10.1002/cncr.28168.

43. Rodríguez-Torres E, Gonzalez-Perez M, Diaz-Perez C et al Barriers and facilitators to the participation of subjects in clinical trials: An overview of reviews. Contemp Clin Trials Commun, 2021; 23:100829. Published 2021 Aug 3. doi:10.1016/j.conctc.2021.100829

44. NIH National Institutes of Health Revitatlization Act of 1993[online] 1993 https://orwh.od.nih.gov/sites/orwh/files/docs/NIH-Revitalization-Act-1993.pdf (accessed 10 Dec 2022)

45. Mays V Research challenges and bioethics responsibilities in the aftermath of the presidential apology to the survivors of the U. S. Public Health Services Syphilis Study at Tuskegee. Ethics & Behavior, 2012; 22(6):419-430. doi:10.1080/10508422.2012.730787

For peer review only - http://bmjopen.bmj.com/site/about/guidelines.xhtml

46. Meinert J, Blehar M, Peindl K et al Bridging the gap: Recruitment of African-American women into mental health research studies. Academic Psychiatry, 2003; 27(1):21-28. doi:10.1176/appi.ap.27.1.21

47. Topp R, Newman J, Jones V et al Including African Americans in health care research. Western Journal of Nursing Research, 2008; 30(2):197-203. doi:10.1177/0193945907303063

48. NIH. Health Report of the Advisory Committee on Research on Women's Health: Fiscal Years 2017–2018 [online] 2019 https://orwh.od.nih.gov/our-work/fiscal-years-2019-2020-full-report-on-research-on-womens-health (accessed 10 Jan 2022).

49. Gilmore-Bykovskyi A, Croff R, Glover C et al Traversing the Aging Research and Health Equity Divide: Toward Intersectional Frameworks of Research Justice and Participation. Gerontologist, 2022; 62(5):711-720. doi:10.1093/geront/gnab107

50. Reihl S, Patil N, Morshed R et al A Population Study of Clinical Trial Accrual for Women and Minorities in Neuro-Oncology Following the NIH Revitalization Act. medRxiv, 2021; 24(8):1341-1349. doi:10.1093/neuonc/noac011

51. Nazha B, Mishra M, Pentz R et al Enrollment of Racial Minorities in Clinical Trials: Old Problem Assumes New Urgency in the Age of Immunotherapy. American Society of Clinical Oncology Educational Book, 2019; 39:3-10. doi:10.1200/EDBK_100021

52. Jiagge E, Kaljee L, Dankerlui D et al Participatory Action for Access to Clinical Trials (PAACT): Increasing participation of blacks in Southeastern Michigan in cancer clinical trails. Journal of Clinical Oncology 2022; 40(16): e18527-e18527 doi: 10.1200/JCO.2022.40.

53. NIHR Equality, Diversity and Inclusion Strategy 2022-2027 [online] 2022 https://www.nihr.ac.uk/documents/equality-diversity-and-inclusion-strategy-2022-2027/31295 (accessed 28 Nov 2022)

54. Jackson J The Challenge of Health Equity in Clinical Research Towards a Quantifiable Science of Inclusion [online] 2020 https://www.nia.nih.gov/research/alzheimers-dementia-outreach-recruitment-engagement-resources/challenge-health-equity (accessed 12 Jan 2023)

55. Ham C Money can't buy you satisfaction. Bmj, 2005; 330(7491):597-599. doi:10.1136/bmj.330.7491.597

56. Díaz Rios L, Stage V, Leak T et al Collecting, Using, and Reporting Race and Ethnicity Information: Implications for Research in Nutrition Education, Practice, and Policy to Promote Health Equity. Journal of Nutrition Education and Behavior, 2022;54(6):582-593. doi:10.1016/j.jneb.2022.01.006

57. National Academies of Sciences, Engineering, and Medicine (NASEM); Health and Medicine Division; Board on Population Health and Public Health Practice; Baciu A, et al eds. Communities in Action: Pathways to Health Equity. Washington (DC): National Academies Press (US) 2017 doi: 10.17226/24624

For peer review only

For peer review only - http://bmjopen.bmj.com/site/about/guidelines.xhtml

58. von Thiele Schwarz U, Nielsen K, Edwards K et al How to design, implement and evaluate organizational interventions for maximum impact: the Sigtuna Principles. European Journal of Work and Organizational Psychology, 2021; 30(3):415-427. Published 2020 Aug 26. doi:10.1080/1359432X.2020.1803960.

59. Pawson, R, Greenhalgh T, Harvey G et al., Realist review--a new method of systematic review designed for complex policy interventions. J Health Serv Res Policy, 2005. 10 Suppl 1: p. 21-34. doi: 10.1258/1355819054308530.

60. Hearst, M, Ekwonye A, Munala L, et al, COVID-19 and Systemic Racism Pandemics Impact Daily Life for Immigrant/Refugees, Minneapolis, USA. Ann Glob Health, 2021. 87(1): p. 107. doi: 10.5334/aogh.3411

61. Devakumar D, Selvarajah S, Shannon G, et al The Lancet Series on racism, xenophobia, discrimination, and health. The Lancet 2022. Jun 27;395(10242):e112-e113. doi: 10.1016/S0140-6736(20)31371-4.

62. Griffiths, S. Weston L, Byng R, et al., Engaging Stakeholders in Realist Programme Theory Building: Insights from the Prospective Phase of a Primary Care Dementia Support Study. International Journal of Qualitative Methods, 2022. 21 doi.org/10.1177/16094069221077521

63. Saul, J. Willis C, Bitz J., et al., A time-responsive tool for informing policy making: rapid realist review. Implementation Science, 2013. 8(1): p. 103. doi.org/10.1186/1748-5908-8-103

64. Pawson, R, Tilley, N Realistic Evaluation. 1997, London: Sage Publications.

65. Pawson R, Greenhalgh T, Harvey G et al Realist review--a new method of systematic review designed for complex policy interventions. J Health Serv Res Policy, 2005; 10 Suppl 1:21-34. doi:10.1258/1355819054308530

66. Wong G,Westhorpe G, Buckingham J et al RAMESES publication standards: realist syntheses. BMC Med., 2013 ;11:21. Published 2013 Jan 29. doi:10.1186/1741-7015-11-21

67. Duddy C ,Wong G Explaining variations in test ordering in primary care: protocol for a realist review. B. BMJ Open, 2018; ;8(9):e023117. Published 2018 Sep 12. doi:10.1136/bmjopen-2018-023117

68. Greenhalgh J , Manzano A Understanding ‘context’ in realist evaluation and synthesis. International Journal of Social Research Methodology, 2022; 25:5, 583-595, doi: 10.1080/13645579.2021.1918484

69. Emmel N, G.J., Manzano A, Monaghan M, et al Doing Realist Research. 2018, London: Sage Publications.

70. Vitae. Research Project Stakeholders [online] 2022 https://www.vitae.ac.uk/doing-research/leadership-development-for-principal-

For peer review only - http://bmjopen.bmj.com/site/about/guidelines.xhtml

investigators-pis/leading-a-research-project/applying-for-research-funding/research-project-stakeholders (accessed 12 Aug 2023)

71. NIHR Guidance on co-producing a research project [online] 2021 https://www.learningforinvolvement.org.uk/content/resource/nihr-guidance-on-co-producing-a-research-project/ (accessed 15 Oct 2022)

72. NIHR Policy Research Unit Older People and Frailty. Experts by Experience [onlin] 2023 https://www.opfpru.nihr.ac.uk/our-people/experts-by-experience/#:~:text=We%20consider%20experts%20by%20experience,patients (accessed 12 Jun 2023)

73. Williams O,Tembo D, Ocloo J et al COVID-19 and Co-production in Health and Social Care Vol 2: Volume 2: Co-production Methods and Working Together at a Distance. Policy Press [online] https://research-portal.uea.ac.uk/en/publications/covid-19-and-co-production-in-health-and-social-care-vol-2-volume (accessed 28 May 2023)

74. Flynn R, Schick-Makaroff K, Greenhalgh J et al Developing an Initial Program Theory to Explain How Patient-Reported Outcomes Are Used in Health Care Settings: Methodological Process and Lessons Learned. International Journal of Qualitative Methods, 2020; 15(1):21-38. doi:10.1007/s40271-021-00530-2

75. Rycroft-Malone J, McCormack B, Hutchinson A et al Realist synthesis: illustrating the method for implementation research. Implementation Science, 2012; 7:33. Published 2012 Apr 19. doi:10.1186/1748-5908-7-33

76. Garden C Lego Serious Play: Building engagement with cell biology. Biochem Mol Biol Educ, 2022; 50(2):216-228. doi:10.1002/bmb.21608,

77. Nielsen M Using artifacts in brainstorming sessions to secure participation and decouple sequentiality. Discourse Studies, 2012. 14(1): 87-109 doi.org/10.1177/1461445611427211 79.

78. Wong G, Greenhalgh T, Pawson R et al Internet-Based medical education : a realist review of what works, for whom and in what circumstances. BMC Med Educ, 2010; 10:12. Published 2010 Feb 2. doi:10.1186/1472-6920-10-1280.

79. Jagosh J CARES Realist Methodology Training [online]. 2022 https://realistmethodology-cares.org/dec2023realistsynthesismasterclass (accessed 10 Dec 2022)

80. Team EndNote. EndNote. 2013; Available from: https://support.clarivate.com/Endnote/s/article/Citing-the-EndNote-program-as-a-reference?language=en_US.(accessed 5Feb 2024)

81. Tacconelli E Systematic reviews:CRD's guidance for undertaking reviews in health care 2010. The Lancet.; doi: :https://doi.org/10.1016/S1473-3099(10)70065-7

For peer review only - http://bmjopen.bmj.com/site/about/guidelines.xhtml

82. Rayyan. Faster Systematic Reviews 2022; Available from: https://www.rayyan.ai/. (accessed 15 Dec 2022)

83. Pawson, R., Evidence-based Policy: A Realist Perspective. 2006, California: Sage

84. Pawson R The Science of Evaluation: A Realist Manifesto. 2013, Los Angeles, California, London, England: Sage 2013;79(3), 580-582 doi.org/10.1177/0020852313497455

85. Wong, G., Westhorp, G., Manzano, A. et al., RAMESES II reporting standards for realist evaluations. BMC Med Educ, 2016; 14(1):96. Published 2016 Jun 24. doi:10.1186/s12916-016-0643-187.

86. Dalkin S, Forster N, Hodgson P et al Using computer assisted qualitative data analysis software (CAQDAS; NVivo) to assist in the complex process of realist theory generation, refinement and testing. International Journal of Social Research Methodology, 2021; 24:1, 123-134,doi: 10.1080/13645579.2020.180352890.

87. Hunter R, Gorely T, Beattie M et al Realist review. International Review of Sport and Exercise Psychology. 2022 15:1,242-265 doi:10.1080/1750984X.2021.1969674

88. Gilmore B, McAuliffe E, Vallieres F et al Data Analysis and Synthesis Within a Realist Evaluation: Toward More Transparent Methodological Approaches. International Journal of Qualitative Methods, 2019; 18: doi.org/10.1177/1609406919859754

89. Jagosh, J, Pluye P, Wong G et al., Critical reflections on realist review: insights from customizing the methodology to the needs of participatory research assessment. Research Synthesis Methods, 2014. 5(2): p. 131-141. doi: 10.1002/jrsm.1099

90. Greenhalgh T, Pawson R, Wong G et al Retroduction in realist evaluation. The RAMESES II Project [online] 2017 http://www.ramesesproject.org/media/RAMESES_II_Retroduction.pdf (accessed 2 Jun 2022).

91. UNISON. Defining Black [online] 2023 https://www.unison.org.uk/about/what-we-do/fairness-equality/black-members/defining-black/ (accessed 26 Jun 2023)

92. Tuckman B Developmental sequence in small groups. Psychological Bulletin, 1965; 63(6), 384–399 doi.org/10.1037/h0022100

For peer review only - http://bmjopen.bmj.com/site/about/guidelines.xhtml

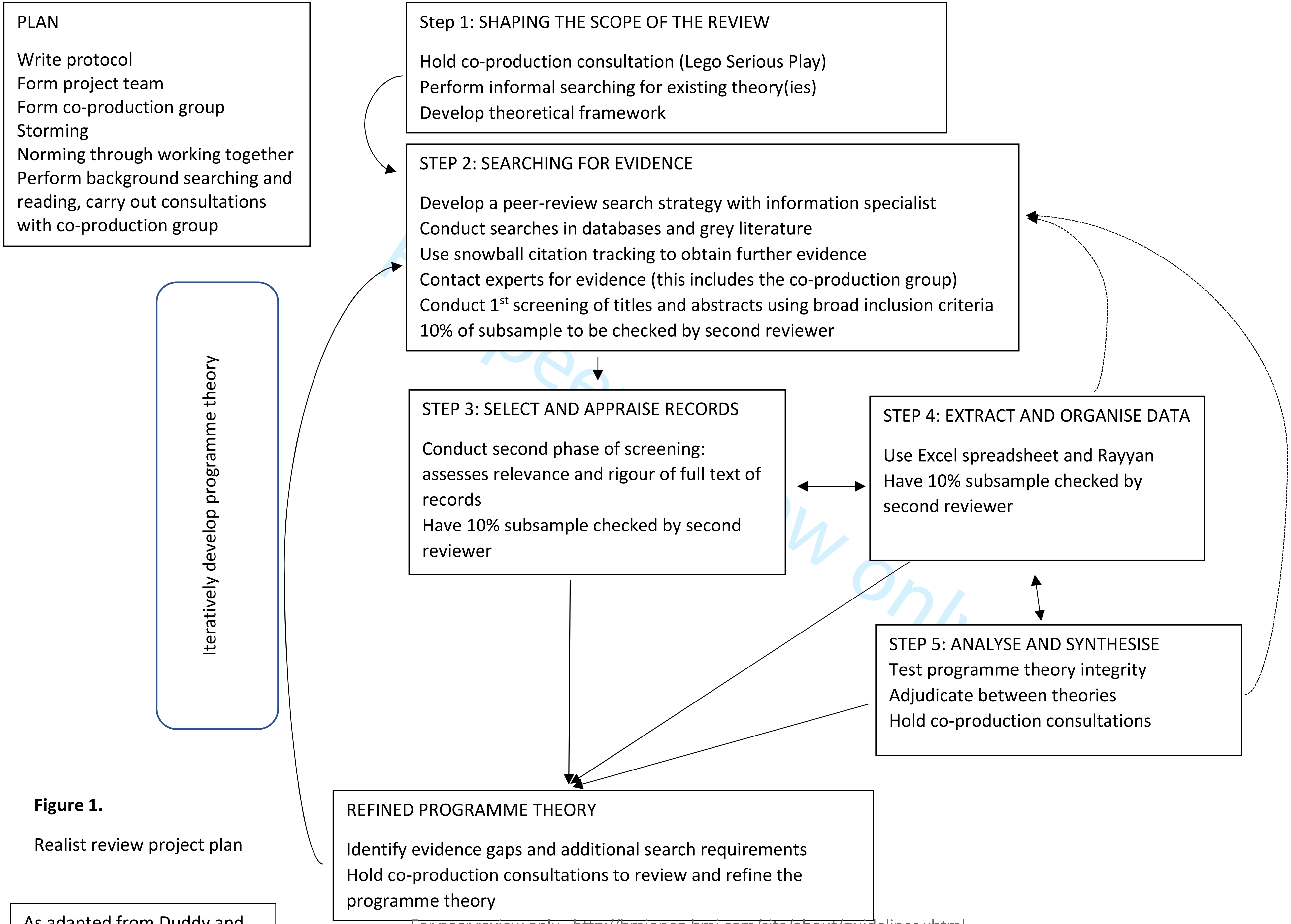

Figure 1.

Realist review project plan

As adapted from Duddy and Wong (2019)

For peer review only - http://bmjopen.bmj.com/site/about/guidelines.xhtml

Figure 2.

Research participation pathway

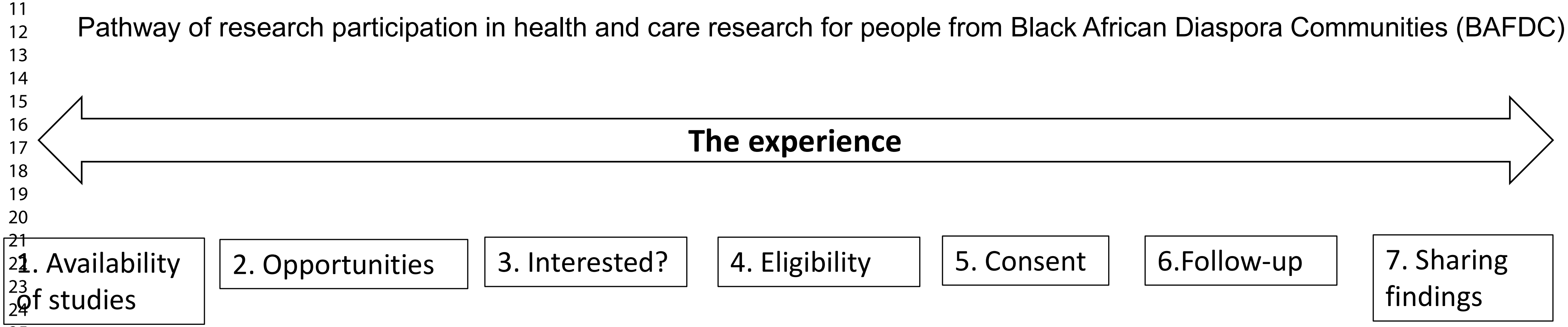

Supplementary file 1. Positionality statement

The authorship team is based in the UK and consists of individuals from the following backgrounds: academic, clinical, clinical academic, leaders of community organisations, health and social care research delivery, health and social care research funding and leaders of third sector organisations, (government and voluntary). All authors have consented to self-reporting their ethnicity, as indicated on the title page. Some have experience of conducting research, delivering research, funding research, providing healthcare, or support and representation in some way, to people from Black African Diaspora Communities (BAFDC). The co-production group (VE, TK, DG, LH, EM,TO, IS, SW) and one member of the research team (DE) have lived experience of being Black, as defined in the protocol. Two members of the co-production group identify as Black men.The first author (EH) is a White British clinical academic researcher whose work mainly focuses on developing an inclusive health and care research system for individuals from BAFDC. Over the last ten years, she has observed the exclusionary practices, processes and service provision of health and social care research to people from BAFDC through her role as a clinical research nurse in the West Midlands region of England. She has been actively involved as a Patient Public Involvement Lead, contributing to her awareness of the impact of exclusion from health and social care research on the health and well-being of people from BAFDC as well as the difficulties they face in opportunities to take part in health and social care research. She and all co-authors acknowledge the importance of equity, diversity and inclusion, and value an inclusive approach to health and social care research where all individuals, including those from under-served communities, benefit from research opportunities. They will be reflexive throughout this research on their positionality and how this may influence interpretations of people's lived experiences. All authors work as a team and the first author (EH) has regular discussions with the co-production group to ensure the protocol and subsequent review will be led by their cultural and experiential knowledge and expertise.

For peer review only

Ovid MEDLINE(R) Example Search Strategy

1	exp Clinical Trials as Topic/ or clinical trial*.mp.
2	exp Health Services Research/ or exp Biomedical Research/
3	exp African Americans/
4	black british.mp.
5	participation.mp. or Patient Participation/ or Community Participation/ or Stakeholder Participation/ or Social Participation/
6	inclusion.mp.
7	recruitment.mp.
8	(underrepresent* or under-represent*).mp.
9	Black people.mp. or Black People/
10	Afro-Caribbean.mp.
11	BME.mp.
12	BAME.mp.
13	1 or 2
14	3 or 4 or 9 or 10 or 11 or 12
15	5 and 13 and
16	6 or 7 or 8
17	5 or 16
18	13 and 14 and 16
19	1 and 5 and 14
20	1 and 14 and 17

For peer review only

PRISMA 2020 Checklist

Section and Topic	Item #	Checklist item	Location where item is reported
**TITLE**			
Title	1	Identify the report as a systematic review. This is a rapid realist review protocol.	Lines 1-3
**ABSTRACT**			
Abstract	2	See the PRISMA 2020 for Abstracts checklist.	Lines 50-78
**INTRODUCTION**			
Rationale	3	Describe the rationale for the review in the context of existing knowledge.	Lines 256-263
Objectives	4	Provide an explicit statement of the objective(s) or question(s) the review addresses.	Lines 296-305
**METHODS**			
Eligibility criteria	5	Specify the inclusion and exclusion criteria for the review and how studies were grouped for the syntheses.	Table 3 (Line 462 ; Line 474)
Information sources	6	Specify all databases, registers, websites, organisations, reference lists and other sources searched or consulted to identify studies. Specify the date when each source was last searched or consulted.	Table 1 Line 418 + Lines 423-430
Search strategy	7	Present the full search strategies for all databases, registers and websites, including any filters and limits used.	Lines 406-421;
Selection process	8	Specify the methods used to decide whether a study met the inclusion criteria of the review, including how many reviewers screened each record and each report retrieved, whether they worked independently, and if applicable, details of automation tools used in the process.	Lines 421-434
Data collection process	9	Specify the methods used to collect data from reports, including how many reviewers collected data from each report, whether they worked independently, any processes for obtaining or confirming data from study investigators, and if applicable, details of automation tools used in the process.	Lines 75-489
Data items	10a	List and define all outcomes for which data were sought. Specify whether all results that were compatible with each outcome domain in each study were sought (e.g. for all measures, time points, analyses), and if not, the methods used to decide which results to collect.	Lines 60-473
	10b	List and define all other variables for which data were sought (e.g. participant and intervention characteristics, funding sources). Describe any assumptions made about any missing or unclear information.	Lines 460-473
Study risk of bias assessment	11	Specify the methods used to assess risk of bias in the included studies, including details of the tool(s) used, how many reviewers assessed each study and whether they worked independently, and if applicable, details of automation tools used in the process.	Lines 462-465<br>Lines 440-452<br>Lines 60-462
Effect measures	12	Specify for each outcome the effect measure(s) (e.g. risk ratio, mean difference) used in the synthesis or presentation of results.	N/A
Synthesis methods	13a	Describe the processes used to decide which studies were eligible for each synthesis (e.g. tabulating the study intervention characteristics and comparing against the planned groups for each synthesis (item #5)).	Lines 491-500
	13b	Describe any methods required to prepare the data for presentation or synthesis, such as handling of missing summary statistics, or data	Lines 491-

For peer review only

PRISMA 2020 Checklist

Section and Topic	Item #	Checklist item	Location where item is reported
		conversions.	500
	13c	Describe any methods used to tabulate or visually display results of individual studies and syntheses.	N/A (protocol)
	13d	Describe any methods used to synthesize results and provide a rationale for the choice(s). If meta-analysis was performed, describe the model(s), method(s) to identify the presence and extent of statistical heterogeneity, and software package(s) used.	N/A (protocol)
	13e	Describe any methods used to explore possible causes of heterogeneity among study results (e.g. subgroup analysis, meta-regression).	N/A (protocol)
	13f	Describe any sensitivity analyses conducted to assess robustness of the synthesized results.	N/A (protocol)
Reporting bias assessment	14	Describe any methods used to assess risk of bias due to missing results in a synthesis (arising from reporting biases).	N/A (protocol)
Certainty assessment	15	Describe any methods used to assess certainty (or confidence) in the body of evidence for an outcome.	N/A (protocol)
**RESULTS**			
Study selection	16a	Describe the results of the search and selection process, from the number of records identified in the search to the number of studies included in the review, ideally using a flow diagram.	No results yet as protocol (417-438 describes search strategy)
	16b	Cite studies that might appear to meet the inclusion criteria, but which were excluded, and explain why they were excluded.	N/A (protocol)
Study characteristics	17	Cite each included study and present its characteristics.	N/A (protocol)
Risk of bias in studies	18	Present assessments of risk of bias for each included study.	N/A (protocol)
Results of individual studies	19	For all outcomes, present, for each study: (a) summary statistics for each group (where appropriate) and (b) an effect estimate and its precision (e.g. confidence/credible interval), ideally using structured tables or plots.	N/A (protocol)
Results of syntheses	20a	For each synthesis, briefly summarise the characteristics and risk of bias among contributing studies.	N/A (protocol)
	20b	Present results of all statistical syntheses conducted. If meta-analysis was done, present for each the summary estimate and its precision (e.g. confidence/credible interval) and measures of statistical heterogeneity. If comparing groups, describe the direction of the effect.	N/A (protocol)
	20c	Present results of all investigations of possible causes of heterogeneity among study results.	N/A (protocol)
	20d	Present results of all sensitivity analyses conducted to assess the robustness of the synthesized results.	N/A (protocol)
Reporting biases	21	Present assessments of risk of bias due to missing results (arising from reporting biases) for each synthesis assessed.	N/A (protocol)

For peer review only

PRISMA 2020 Checklist

Section and Topic	Item #	Checklist item	Location where item is reported
Certainty of evidence	22	Present assessments of certainty (or confidence) in the body of evidence for each outcome assessed.	N/A (protocol)
**DISCUSSION**			
Discussion	23a	Provide a general interpretation of the results in the context of other evidence.	N/A (protocol)
	23b	Discuss any limitations of the evidence included in the review.	N/A (protocol)
	23c	Discuss any limitations of the review processes used.	Lines 509-512
	23d	Discuss implications of the results for practice, policy, and future research.	Lines 275-278; 288-295
**OTHER INFORMATION**			
Registration and protocol	24a	Provide registration information for the review, including register name and registration number, or state that the review was not registered.	Not registered
	24b	Indicate where the review protocol can be accessed, or state that a protocol was not prepared.	N/A (protocol)
	24c	Describe and explain any amendments to information provided at registration or in the protocol.	N/A (protocol)
Support	25	Describe sources of financial or non-financial support for the review, and the role of the funders or sponsors in the review.	Lines 552-558
Competing interests	26	Declare any competing interests of review authors.	Line 559
Availability of data, code and other materials	27	Report which of the following are publicly available and where they can be found: template data collection forms; data extracted from included studies; data used for all analyses; analytic code; any other materials used in the review.	N/A (protocol)

From: Page MJ, McKenzie JE, Bossuyt PM, Boutron I, Hoffmann TC, Mulrow CD, et al. The PRISMA 2020 statement: an updated guideline for reporting systematic reviews. BMJ 2021;372:n71. doi: 10.1136/bmj.n71

For more information, visit: http://www.prisma-statement.org/

For peer review only
